# Long-Term Outcomes of Nephron-Sparing Versus Radical Nephrectomy in Stage 4 Chronic Kidney Disease

**DOI:** 10.3390/jcm14227951

**Published:** 2025-11-10

**Authors:** Nai-Wen Chang, Huan-Nung Chao, Chia-Ying Yu, Ya-Chuan Chang, Sung-Lang Chen, Tzuo-Yi Hsieh, Wen-Wei Sung

**Affiliations:** 1 School of Medicine, Chung Shan Medical University, Taichung 40201, Taiwan; cshy1865@csh.org.tw (N.-W.C.); cshn948@csh.org.tw (C.-Y.Y.); cshn947@csh.org.tw (Y.-C.C.); cshy650@csh.org.tw (S.-L.C.); 2 Department of Urology, Chung Shan Medical University Hospital, Taichung 40201, Taiwan; 3 Division of Nephrology, Department of Internal Medicine, Hanming Christian Hospital, Changhua 50058, Taiwan; 1310008@cch.org.tw; 4 Institute of Medicine, Chung Shan Medical University, Taichung 40201, Taiwan

**Keywords:** chronic kidney disease, cryotherapy, electrolyte imbalance, end-stage renal disease, nephrectomy, renal function

## Abstract

**Background:** Partial nephrectomy (PN) and radical nephrectomy (RN) are surgical options for localized renal cell carcinoma; however, PN is p referred for preserving renal function in patients with chronic kidney disease (CKD). This study compares the risks of end-stage renal disease (ESRD), hemodialysis, and cardiovascular complications between PN and RN in patients with stable stage 4 CKD, with a further focus on postoperative electrolyte imbalances as outcome predictors. **Methods:** This retrospective cohort study used TriNetX data (between 2005 and 2023). Patients with stable stage 4 CKD undergoing nephron-sparing procedures or RN were included. Propensity-score matching ensured balanced baseline characteristics. The analyzed outcomes included renal function, hemodialysis, electrolyte imbalances, and cardiovascular events. Statistical analyses were hazard ratios (HRs) and risk ratios (RRs) with 95% confidence intervals (CIs). **Results:** The PN and RN groups showed no statistically significant differences in their progression to ESRD or need for long-term hemodialysis. Patients undergoing RN had a higher risk of pulmonary embolism (HR: 2.60; 95% CI: 1.12–6.02). Electrolyte imbalances, particularly abnormal calcium and phosphate levels, were more common in the RN cohort in the early postoperative period, but they stabilized over time. Notably, these electrolyte imbalances were associated with an increased risk of cardiovascular complications. The study limitations include the absence of tumor grade, tumor staging, and pathological information. **Conclusions:** Nephron-sparing PN did not reduce ESRD risk compared with RN, but provided a lower incidence of certain postoperative complications, such as pulmonary embolism and early electrolyte disturbances. Early postoperative electrolyte imbalances, especially abnormal calcium and phosphate levels, may predict adverse renal and cardiovascular outcomes.

## 1. Introduction

For the treatment of localized renal cell carcinoma, surgical options include partial nephrectomy (PN) and radical nephrectomy (RN). Other primary treatment options include active surveillance and ablative therapies for selected patients [1,2,3,4]. For small renal tumors, both the nephron-sparing PN and radical RN surgeries have comparable oncological outcomes, as demonstrated by the EORTC-30904 European randomized trial [1]. In general, PN is recommended for individuals at risk of developing progressive chronic kidney disease (CKD) [2,3]. To preserve renal function, the European Association of Urology (EAU) guidelines for renal cell carcinoma (RCC) advocate the use of PN in patients with CKD and localized tumors [4], and the AUA guidelines similarly prioritize nephron-sparing approaches for patients with CKD [5]. Previous studies, including systematic reviews, have suggested a lower risk of progression to severe CKD after PN [3,6,7,8]. However, most of these studies have focused on populations with baseline normal renal function in early-stage CKD and have analyzed the progression to severe CKD and end-stage renal disease (ESRD) after extirpative surgery.

A recent study reported no significant difference in the risk of progression to ESRD following PN or RN in patients with preoperative severe CKD [9]. This finding suggests that the choice of surgical approach may have less impact on ESRD progression in this population than previously anticipated. However, patients with CKD remain at a higher risk for cardiovascular disease and associated mortality [10,11,12], and impaired renal function following surgery has also been linked to cardiovascular-specific survival [13,14]. Furthermore, electrolyte imbalances are also common in patients with CKD, according to the limited number of studies that have examined changes in electrolyte levels after nephrectomy [15,16]. These potential cardiovascular and electrolyte disturbances following nephrectomy suggest that surgical strategies for patients with CKD still require further augmentation.

Recent updates in renal cancer management underscore the importance of optimizing surgical strategies for patients with advanced CKD. The EAU and National Comprehensive Cancer Network (NCCN) guidelines highlight the need to balance oncological control with renal preservation when selecting between nephron-sparing PN and radical RN procedures [2,4]. Several contemporary studies have advanced this idea by integrating minimally invasive and ablative approaches and demonstrating comparable oncological safety with improved perioperative outcomes [3,17]. Emerging evidence has also revealed that postoperative metabolic and electrolyte disturbances may serve as early indicators of renal recovery and cardiovascular risk in CKD populations. Collectively, these recent findings strengthen the rationale for the present study, which explores the association between early biochemical alterations and long-term renal and systemic outcomes in patients with stage 4 CKD.

Data for patients with preoperative severe CKD remain scarce regarding their progression to ESRD, hemodialysis dependency, and cardiovascular morbidity and mortality. Therefore, the aim of this study was to compare nephron-sparing PN techniques and RN in terms of the risks of adverse outcomes by investigating electrolyte changes, ESRD progression, hemodialysis requirements, and cardiovascular morbidity in patients with preoperative stage 4 CKD. We also investigated whether early postoperative electrolyte imbalances might predict adverse renal and systemic outcomes in these patients and potentially serve as early indicators that could mitigate CKD progression and related complications.

## 2. Methods

### 2.1. Data Availability Statement

The patient data used in this study were obtained from TriNetX (https://trinetx.com, accessed on 23 July 2025), a global federated health research network that provides real-time access to anonymized electronic medical records from over 120 healthcare organizations worldwide. No identifiable patient information is included in this article. The TriNetX platform aggregates de-identified patient-level data, including demographics, diagnoses, procedures, laboratory results, and medications, in accordance with HIPAA (Health Insurance Portability and Accountability Act) standards. No identifiable patient information is included in this article, and no data extraction outside the platform was performed.

### 2.2. Study Design and Patient Selection

This was a retrospective cohort study using data from the US Collaborative Network of the TriNetX database. The data from the US Collaborative Network includes contributions of data from 67 healthcare organizations obtained from 1 January 2005 to 31 December 2023.

**Figure 1:**
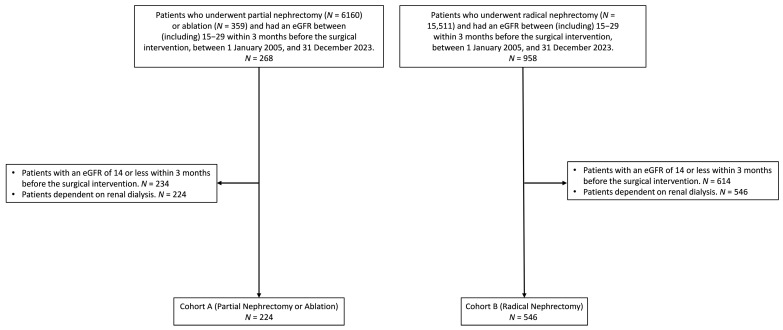
Cohort selection process for patients with stage 4 chronic kidney disease who underwent partial nephrectomy, ablation, or radical nephrectomy between 1 January 2005, and 31 December 2023. Patients with an eGFR ≤ 14 within 3 months before surgery or dependence on renal dialysis were excluded. The final cohorts, before matching, included 224 patients in Cohort A (partial nephrectomy or ablation) and 546 patients in Cohort B (radical nephrectomy).

Two cohorts were defined based on patient data and procedure details, as shown in Figure 1. Cohort A consisted of patients with stage 4 CKD who underwent nephron-sparing procedures, including PN or renal mass ablation, and who met similar inclusion criteria. Patients with a GFR ≤ 14.0 mL/min/1.73 m^2^ or dependence on hemodialysis prior to surgery were excluded. Cohort B included patients with stage 4 CKD who underwent RN, and met the inclusion criterion of a glomerular filtration rate (GFR) between 15.0 and 29.0 mL/min/1.73 m^2^ within three months before the procedure. Follow-up continued until death, hemodialysis initiation, or 12 months after the index procedure, whichever came earliest. Demographics, comorbidities, and laboratory variables were extracted for adjustment and matching. Propensity score matching, applied to balance the baseline characteristics between the cohorts, accounted for demographics, comorbidities, and laboratory values to minimize potential confounding.

The outcomes assessed in this study included electrolyte imbalances, which were analyzed across the following time intervals: 3 days–1 week, 1 week–1 month, 1 month–3 months, 3 months–6 months, and 6 months–1 year. The analysis examined worsening eGFR, serum creatinine levels, and the initiation of hemodialysis during the follow-up period. Overall cardiovascular and skeletal events were also evaluated, including acute myocardial infarction (ICD-10: I21), pulmonary embolism (ICD-10: I26), cardiac arrest (ICD-10: I46), nontraumatic cerebral hemorrhage (ICD-10: I60–I62), cerebral infarction (ICD-10: I63), unspecified peripheral vascular disease (ICD-10: I73.9), acute embolism and thrombosis of deep veins of the lower extremities (ICD-10: I82.4), osteoporosis with current pathological fracture (ICD-10: M80), and osteoporosis without current pathological fracture (ICD-10: M81). These outcomes were analyzed to evaluate the systemic and skeletal complications associated with nephron-sparing approaches and RN in patients with severe CKD. All outcome definitions were based on ICD-10 codes predefined in the TriNetX terminology library to ensure cross-institutional consistency.

### 2.3. Ethics Statement

The TriNetX platform complies with the Health Insurance Portability and Accountability Act (HIPAA) and the General Data Protection Regulation (GDPR). The Western Institutional Review Board (WIRB) granted a waiver to TriNetX, as it provides only aggregated counts and statistical summaries of anonymized data. The use of TriNetX for this study was approved by the Institutional Review Board of Chung Shan Medical University Hospital (CSMUH No: CS1-24177, Date: 26 November 2024). All analyses were conducted in accordance with the tenets of the Declaration of Helsinki (2024).

### 2.4. Statistical Analysis

All statistical analyses were performed using the TriNetX analytics platform. The effect of confounding factors was minimized using the built-in propensity score generation feature of TriNetX to perform 1:1 matching and greedy nearest neighbor matching with a caliper of 0.1 pooled standard deviations between the two groups. Standardized mean differences (SMDs) were calculated to compare the two groups before and after matching, with an SMD below 0.1 considered to indicate good balance. HRs or RRs, along with their associated CIs, were calculated, and the proportionality assumption was tested. Continuous variables were expressed as mean ± SD and compared using Student’s *t*-test, while categorical variables were compared using the χ^2^ test. Kaplan–Meier survival curves were generated to compare time-to-event outcomes, and differences were assessed using the log-rank test. The proportional hazards assumption was examined for all Cox models. Survival curve differences between groups were assessed using the log-rank test, which was also performed within TriNetX. A *p*-value of <0.05 was considered statistically significant.

## 3. Results

### 3.1. Cohort Selection and Characteristics

A total of 268 patients who underwent PN or ablation and 958 patients who underwent RN were initially identified based on their eGFR values of 15–29 mL/min/1.73 m^2^ in the 3-month period prior to surgery. Patients with an eGFR ≤ 14 in the 3 months prior to surgery or those dependent on renal dialysis were excluded, leaving 224 patients in Cohort A (PN or ablation) and 546 patients in Cohort B (RN) (Figure 1). Table 1 presents the demographic and clinical characteristics before and after matching between the cohorts. After propensity score matching, 219 patients remained in each cohort, with no significant differences in age, race, BMI, or underlying diseases, as evidenced by SMDs approaching zero. The median follow-up time was 1586 days (interquartile range, 2195 days) for patients in Cohort A and 1063 days (interquartile range, 2346 days) for patients in Cohort B.

### 3.2. Risk of Electrolyte and Metabolic Abnormalities Across Postoperative Time Intervals

**Figure 2:**
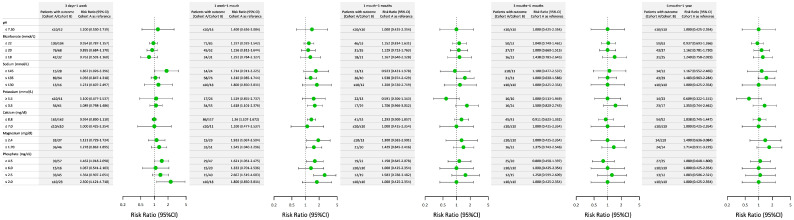
Comparison of the risk ratios for electrolyte and metabolic abnormalities between Cohort A (partial nephrectomy or ablation) and Cohort B (radical nephrectomy) across various postsurgical time intervals. This figure illustrates the risk ratios (RRs) and corresponding 95% confidence intervals (CIs) for electrolyte and metabolic abnormalities between Cohort A and Cohort B. The data are stratified across five postoperative time intervals: 3 days–1 week, 1 week–1 month, 1 month–3 months, 3 months–6 months, and 6 months–1 year. The outcomes include abnormalities in pH, bicarbonate, sodium, potassium, calcium, magnesium, and phosphate levels. Each dot represents the RR with its 95% CI, with Cohort A (partial nephrectomy or ablation) serving as the reference group.

Figure 2 compares the risk ratios (RRs) of electrolyte and metabolic abnormalities between the PN and RN cohorts over five postoperative time intervals. In the early postoperative period (3 days–1 week), abnormalities in sodium (≥145 mmol/L) and phosphate (≥4.5 and ≤2.0 mg/dL) levels showed higher risk trends in Cohort B than in Cohort A. Between 1 week and 1 month, phosphate abnormalities persisted, with patients in Cohort B showing significantly higher risks for phosphate levels ≥4.5 and ≤2.5 mg/dL. Potassium (≤3.5 mmol/L), calcium (≤8.8 mg/dL), and magnesium (≥2.4 and ≤1.7 mg/dL) levels also showed elevated risks. At 1 month–3 months, differences in RRs narrowed for most electrolyte parameters. However, the elevated risk for phosphate abnormalities (RR: 1.64; 95% CI: 1.09–2.55) remained more pronounced in Cohort B. For other parameters, including sodium and pH, the risks appeared similar between the two cohorts. In the 3-month–6-month and 6-month–1-year intervals, the RRs for most abnormalities stabilized across both cohorts. Overall, the findings suggested that patients in Cohort B had transiently higher risks of electrolyte and metabolic abnormalities in the early postoperative period, particularly within the first month, whereas the other electrolyte imbalances stabilized over time.

### 3.3. Renal Function and Long-Term CKD-Related Cardiovascular and Skeletal Outcomes

**Figure 3:**
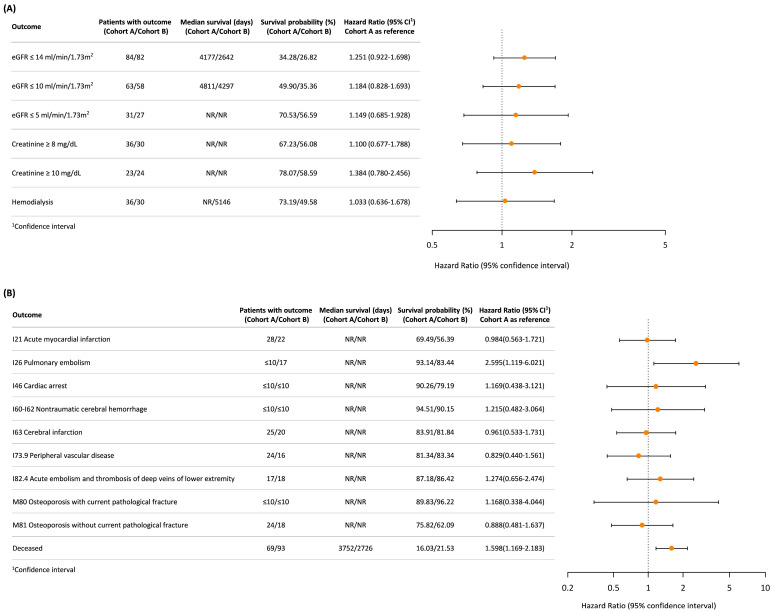
Comparison of renal function outcomes and long-term vascular and skeletal events between (**A**) Cohort A (partial nephrectomy or ablation) and (**B**) Cohort B (radical nephrectomy). Each dot represents the hazard ratio (HR) with its 95% confidence interval (CI).

Figure 3 compares renal function outcomes, cardiovascular events, skeletal events, and crude overall mortality between cohorts. For the renal function outcomes (Figure 3A), the hazard ratios (HRs) for all outcomes were generally higher in Cohort B, although the differences were not statistically significant. The need for hemodialysis also showed no significant difference between the PN and RN cohorts (HR: 1.033; 95% CI: 0.636–1.678). For the cardiovascular and skeletal events (Figure 3B), Cohort B showed a significantly higher risk of pulmonary embolism (HR: 2.595; 95% CI: 1.119–6.021), while the risks for other events, including skeletal events, were similar between both groups, even though the crude overall mortality was higher in Cohort B.

This figure summarizes the hazard ratios (HRs), survival probabilities (%), and median survival days for renal function outcomes (Panel A) and long-term cardiovascular and skeletal events (Panel B) between the two cohorts. Panel A focuses on renal outcomes, including eGFR thresholds, creatinine levels, and hemodialysis dependence. Panel B presents the outcomes for long-term cardiovascular events. The risk of crude mortality (deceased) is also depicted.

### 3.4. Electrolyte Imbalances Between 1 Week and 1 Month as Predictors of Renal and Long-Term Outcomes

We investigated whether electrolyte imbalances occurring between 1 week and 1 month postoperatively could serve as predictors of subsequent renal and CKD-related outcomes by combining all patients, regardless of surgical intervention type, for further analysis (Supplementary Figure S1A, *N* = 1176). The cutoff values for the abnormal electrolyte values are summarized in Supplementary Table S1, while the demographic and clinical characteristics before and after matching between the cohorts are summarized in Supplementary Tables S2–S8.

Supplementary Figure S1B reveals that patients with abnormal calcium levels also showed increased risks of pulmonary embolism (HR: 5.486; 95% CI: 2.275–13.230). Abnormal phosphate levels were strongly associated with adverse outcomes, including a significantly elevated risk for severe renal impairment, hemodialysis, cerebral infarction, and acute embolism and thrombosis of deep veins of the lower extremities (HR: 3.374; 95% CI: 1.732–6.570). Meanwhile, abnormal sodium and potassium levels exhibited varying degrees of association with long-term outcomes, although these trends were less pronounced. In summary, electrolyte imbalances measured between postoperative 1 week and 1 month were associated with worse renal and long-term outcomes and may be critical for predicting and mitigating CKD progression and related complications. Detailed results are summarized in Supplementary Figures S1–S7.

## 4. Discussion

Our study is the first to compare the electrolyte level, progression to ERSD, need for hemodialysis, and cardiovascular morbidity and overall mortality between nephron-sparing approaches and RN in patients with preexisting stage 4 CKD. Our work uniquely addresses a critical knowledge gap by focusing on patients with stable stage 4 CKD—a population often underrepresented in nephrectomy research. By comparing the long-term risks of ESRD, hemodialysis dependency, and cardiovascular complications between PN and RN, we provide valuable insights into surgical decision making for high-risk patients. Moreover, we highlight the predictive role of early postoperative electrolyte imbalances, particularly abnormal calcium and phosphate levels, in identifying patients at an increased risk of adverse renal and cardiovascular outcomes. Although our results are similar to those reported in a recent study, Cohort A includes patients receiving PN and ablative therapies [9]. Furthermore, we extend the previous conclusion to report no difference in ESRD and hemodialysis between nephron-sparing approaches, such as ablative therapy, and RN.

Comparing nephron-sparing approaches, including ablative therapies, with RN is more in line with the decisions faced by clinical patients. Ablative therapy through the percutaneous technique is an alternative treatment that minimizes morbidity when treating elderly patients with multiple comorbidities or anxiety about surgery [5]. Similarly, initial clinical experiences with novel robotic systems, such as Senhance, have demonstrated the feasibility and safety of ablative procedures in urologic oncology, thereby broadening the minimally invasive options for high-risk patients with CKD [17]. Notably, a systemic review has shown no significant difference in renal function outcomes between PN and thermal ablation [18,19].

Regarding cardiovascular morbidity, most studies have demonstrated that PN decreases cardiovascular risk compared to RN [14,20,21]. Another propensity score study revealed no difference in the likelihood of adverse cardiovascular outcomes between the two surgical approaches [22]. However, among all the available studies, the baseline renal function of the populations was comparable to that of our population. Several studies have demonstrated a higher risk of CKD progression for medical CKD than for surgical CKD; moreover, they have shown that a more severe CKD has higher cardiovascular morbidities [13,21,23]. Considering the worse renal function of our patients, the finding of no difference in cardiovascular morbidities between the two cohorts (apart from a higher rate of pulmonary embolism in Cohort B) was reasonable. However, we did not analyze the number of benign and renal cell carcinomas between the two cohorts, nor did we record the associated history, including cancer and venous thromboembolism. The proportion of renal cell carcinomas may have been higher in Cohort B than in Cohort A, and because nephrectomy is a standard treatment for renal cell carcinoma, this could explain the higher pulmonary embolism rate in Cohort B.

Likewise, regarding the higher overall mortality in Cohort B, we did not record the details of the renal cell carcinomas, including their pathological stages and tumor grades. The tumor stages and grades may have been higher in Cohort B than in Cohort A, which could be the cause of the higher overall mortality in Cohort B.

Electrolyte imbalances could also have increased the mortality rate [24]. Our study is the first to analyze the electrolyte levels after nephron-sparing approaches and RN. We observed that a risk difference for an electrolyte imbalance between the two cohorts occurs mainly at 3 days–1 week and 1 week–1 month, but the risk stabilizes after one month. The causes of electrolyte imbalances in postoperative patients can be multifactorial, including blood loss during surgery, the amount of drainage, insufficient fluid management, and medication usage. A previous study revealed that among urological operations, kidney operations had higher rates of postoperative hyponatremia and were significantly associated with postoperative hyponatremia [25]. Sodium and water balances are critical issues in patients with advanced CKD [26]. We did not find a significant difference at 1 week postoperatively; however, we observed that patients in Cohort B had a higher risk of hyperphosphatemia (*p* ≥ 4.5 mg/dL) in the early postoperative period (3 days–1 week and 1 week–1 month).

Hyperphosphatemia is a pathophysiological consequence of CKD [27]. We speculate that the temporary postoperative renal injury and loss of renal function in Cohort B interfered with electrolyte regulation. Our analysis of the risk of mortality and cardiovascular morbidities based on different electrolyte imbalances revealed that patients with postoperatively abnormal calcium and phosphate levels had a higher risk of certain cardiovascular morbidities, which may be related to vascular calcification. Our results are compatible with the findings from previous observational studies [28,29,30].

However, our study has some limitations. One is that we did not present the characteristics of the renal tumors, including benign and renal cell carcinomas, tumor size, pathology, and grade. Other limitations are that the nephron-sparing group combined partial nephrectomy and ablation, which are distinct procedures, and that the study lacked a detailed follow-up duration, potentially affecting interpretation and statistical robustness. We also did not present the preoperative electrolyte levels of our population. Another limitation is that the individual trajectories of electrolyte changes could not be assessed, as the TriNetX database only provides aggregated rather than patient-level longitudinal data. We also did not include perioperative data, such as blood loss or operative time. Furthermore, the postoperative intervals of renal function were not recorded, which could have impacted some electrolyte changes. The split renal function was also not presented. The long enrollment period (approximately 20 years) may introduce potential bias, as surgical techniques and perioperative management strategies have evolved substantially since 2005. Future studies focusing on patients treated within the past decade would help minimize temporal bias and better reflect current clinical practice. Finally, differences in postoperative care in the hospitals could have influenced the renal function outcomes and patient survival.

## 5. Conclusions

This study compares the risks of ESRD and hemodialysis dependency following nephron-sparing and RN surgeries in patients with stable stage 4 CKD. Overall, our findings underscore the complexity of surgical decision making in this vulnerable population. The study emphasizes the role of early postoperative electrolyte imbalances, particularly abnormal calcium and phosphate levels, in predicting adverse renal and cardiovascular outcomes. Our findings suggest that nephron-sparing approaches may mitigate certain postoperative complications without increasing long-term renal risks, thereby offering critical insights for optimizing patient management. Further research is warranted to refine strategies for postoperative care and to improve outcomes in high-risk patients with CKD. Importantly, our study provides a novel perspective by linking early postoperative electrolyte disturbances to long-term renal and cardiovascular outcomes in patients with advanced CKD—a relationship rarely explored in previous nephrectomy research. However, our study’s limitations include a lack of detailed tumor characteristics, perioperative parameters, and preoperative electrolyte profiles, as well as sidestepping the possible role of variations in postoperative care among institutions. Future multicenter prospective studies that integrate pathological, biochemical, and longitudinal clinical data are needed to validate our findings and elucidate the underlying mechanisms and consequences of electrolyte dysregulation following nephrectomy.

## Figures and Tables

**Table 1:** Demographic and clinical characteristics before and after matching between Cohort A (partial nephrectomy or ablation) and Cohort B (radical nephrectomy).

Attribute	Before Matching				After Matching			
	Cohort A (Partial Nephrectomy or Ablation)	Cohort B (Radical Nephrectomy)			Cohort A (Partial Nephrectomy or Ablation)	Cohort B (Radical Nephrectomy)		
	*N* = 224	*N* = 546			*N* = 219	*N* = 219		
Demographics	*N* (%)	*N* (%)	*p*-value	SMD ^2^	*N* (%)	*N* (%)	*p*-value	SMD ^2^
Age (years)								
Current Age	74.0 ± 11.7	71.0 ± 14.3	0.007	0.223	74.0 ± 11.7	75.2 ± 11.4	0.297	0.100
Age at Index	65.3 ± 11.8	63.6 ± 14.2	0.104	0.134	65.5 ± 11.9	65.8 ± 12.1	0.793	0.025
Gender								
Male	130 (58.0)	311 (57.0)	0.784	0.022	126 (57.5)	127 (58.0)	0.923	0.009
Female	92 (41.1)	220 (40.3)	0.842	0.016	91 (41.6)	92 (42.0)	0.923	0.009
Unknown Gender ^1^	≤10 (≤4.5)	14 (2.7)	--	--	≤10 (≤4.6)	0 (0.0)	--	--
Ethnicity								
Not Hispanic or Latino	159 (71.0)	380 (69.6)	0.703	0.030	156 (71.2)	151 (68.9)	0.602	0.050
Hispanic or Latino	19 (8.5)	53 (9.7)	0.596	0.043	18 (8.2)	18 (8.2)	1.000	<0.001
Unknown Ethnicity	46 (20.5)	113 (20.7)	0.960	0.004	45 (20.5)	50 (22.8)	0.562	0.055
Race								
White	143 (63.8)	405 (74.2)	0.004	0.225	142 (64.8)	145 (66.2)	0.763	0.029
Asian ^1^	≤10 (≤4.5)	≤10 (≤1.8)	0.037	0.151	≤10 (≤4.6)	≤10 (≤4.6)	1.000	<0.001
Black or African American	38 (17.0)	66 (12.1)	0.072	0.139	37 (16.9)	39 (17.8)	0.801	0.024
American Indian or Alaska Native ^1^	≤10 (≤4.5)	0 (0.0)	<0.001	0.306	0 (0.0)	0 (0.0)	--	--
Other Race ^1^	≤10 (≤4.5)	≤10 (≤1.8)	0.037	0.151	≤10 (≤4.6)	≤10 (≤4.6)	1.000	<0.001
Unknown Race	28 (12.5)	57 (10.4)	0.407	0.065	27 (12.3)	26 (11.9)	0.884	0.014
Nicotine dependence	23 (10.3)	59 (10.8)	0.826	0.018	22 (10.0)	22 (10.0)	1.000	<0.001
BMI (kg/m^2^)	30.7 ± 7.8		0.567	0.051	30.6 ± 7.9	30.1 ± 7.9	0.501	0.072
–18.5 kg/m^2 1^	11 (4.9)	28 (5.1)	0.901	0.010	11 (5.0)	≤10 (≤4.6)	0.823	0.021
18.5–25 kg/m^2^	56 (25.0)	130 (23.8)	0.726	0.028	56 (25.6)	58 (26.5)	0.828	0.021
25–30 kg/m^2^	88 (39.3)	213 (39.0)	0.943	0.006	85 (38.8)	89 (40.6)	0.696	0.037
30– kg/m^2^	101 (45.1)	237 (43.4)	0.669	0.034	98 (44.7)	93 (42.5)	0.630	0.046
eGFR								
15–20 mL/min/{1.73_m^2^}	60 (26.8)	158 (28.9)	0.547	0.048	59 (26.9)	61 (27.9)	0.830	0.020
20–25 mL/min/{1.73_m^2^}	103 (46.0)	282 (51.6)	0.153	0.114	101 (46.1)	112 (51.1)	0.293	0.101
25–30 mL/min/{1.73_m^2^}	149 (66.5)	395 (72.3)	0.107	0.127	147 (67.1)	153 (69.9)	0.537	0.059
Underlying disease								
Diabetes mellitus	84 (37.5)	225 (41.2)	0.340	0.076	82 (37.4)	84 (38.4)	0.844	0.019
Hypertensive diseases	185 (82.6)	426 (78.0)	0.155	0.115	180 (82.2)	174 (79.5)	0.466	0.070
Heart failure	33 (14.7)	95 (17.4)	0.367	0.073	32 (14.6)	35 (16.0)	0.690	0.038
Cerebrovascular diseases	33 (14.7)	76 (13.9)	0.769	0.023	33 (15.1)	26 (11.9)	0.327	0.094
Ischemic heart diseases	60 (26.8)	174 (31.9)	0.164	0.112	59 (26.9)	62 (28.3)	0.749	0.031
Dyslipidemia	129 (57.6)	272 (49.8)	0.050	0.156	125 (57.1)	122 (55.7)	0.773	0.028

^1^ To safeguard patients’ protected health information (PHI), 1 to 9 cases were rounded up to 10. ^2^ Standardized Mean Difference.

## Data Availability

Data used in this analysis are de-identified and publicly available on the TriNetX website (https://trinetx.com/, accessed on 23 July 2025). Data are available to bona fide researchers who request it from the authors.

## Supplementary Materials

The following supporting information can be downloaded at: https://www.mdpi.com/article/10.3390/jcm14227951/s1. Table S1: Cutoff values for abnormal electrolyte levels detected between 1 week and 1 month postoperatively. Table S2. Demographic and clinical characteristics of patients with blood pH > 7.3 and pH ≤ 7.3, assessed between 1 week and 1 month postoperatively, both before and after matching. Table S3. Demographic and clinical characteristics of patients with blood HCO_3^−^_ > 22 and HCO_3^−^_ ≤ 22 (mmol/L), assessed between 1 week and 1 month postoperatively, both before and after matching. Table S4. Demographic and clinical characteristics of patients with blood Na^+^ wnl and Na^+^ abn, assessed between 1 week and 1 month postoperatively, both before and after matching. Table S5. Demographic and clinical characteristics of patients with blood K^+^ wnl and K^+^ abn, assessed between 1 week and 1 month postoperatively, both before and after matching. Table S6. Demographic and clinical characteristics of patients with blood Ca^2+^ wnl and Ca^2+^ abn, assessed between 1 week and 1 month postoperatively, both before and after matching. Table S7. Demographic and clinical characteristics of patients with blood Mg^2+^ wnl and Mg^2+^ abn, assessed between 1 week and 1 month postoperatively, both before and after matching. Table S8. Demographic and clinical characteristics of patients with blood P wnl and P abn, assessed between 1 week and 1 month postoperatively, both before and after matching. Figure S1. Comparison of renal function and long-term outcomes between patients with normal and abnormal electrolyte parameters measured between 1 week and 1 month, regardless of surgical intervention type. Figure S2. Comparison of renal function outcomes and long-term vascular and skeletal events between patients with blood pH > 7.3 and pH ≤ 7.3, assessed between 1 week and 1 month postoperatively. Figure S3. Comparison of renal function outcomes and long-term vascular and skeletal events between patients with blood HCO_3^−^_ > 22 and HCO_3^−^_ ≤ 22 (mmol/L), assessed between 1 week and 1 month postoperatively. Figure S4. Comparison of renal function outcomes and long-term vascular and skeletal events between patients with blood Na+ wnl and Na+ abn, assessed between 1 week and 1 month postoperatively. Figure S5. Comparison of renal function outcomes and long-term vascular and skeletal events between patients with blood K^+^ wnl and K^+^ abn, assessed between 1 week and 1 month postoperatively. Figure S6. Comparison of renal function outcomes and long-term vascular and skeletal events between patients with blood Ca^2+^ wnl and Ca^2+^ abn, assessed between 1 week and 1 month postoperatively. Figure S7. Comparison of renal function outcomes and long-term vascular and skeletal events between patients with blood Mg^2+^ wnl and Mg^2+^ abn, assessed between 1 week and 1 month postoperatively. Figure S8. Comparison of renal function outcomes and long-term vascular and skeletal events between patients with blood P wnl and P abn, assessed between 1 week and 1 month postoperatively.
