# Supplementary material for: Long-Term Outcomes of Nephron-Sparing Versus Radical Nephrectomy in Stage 4 Chronic Kidney Disease
Source: J Clin Med. 2025 Nov 10;14(22):7951. doi: 10.3390/jcm14227951 (PMC12653262; doi:10.3390/jcm14227951)
Supplement: Supplementary file 1 [file jcm-14-07951-s001.zip › jcm-3920081-supplementary.pdf]

Supplementary Table 1. Cutoff values for abnormal electrolyte levels detected between 1 week and 1 month postoperatively.

Parameter	wnl	abn	unit
Na^{+}	134-144	≤ 135 or ≥ 145	mmol/L
K^{+}	3.4-5.4	≤ 3.5 or ≥ 5.5	mmol/L
Ca^{2+}	>8.8	≤ 8.8	mg/dL
Mg^{2+}	1.8-2.3	≤ 1.7 or ≥ 2.4	mg/dL
P	2.6-4.4	≤ 2.5 or ≥ 4.5	mg/dL

Supplementary Table 2. Demographic and clinical characteristics of patients with blood pH > 7.3 and pH ≤ 7.3, assessed between 1 week and 1 month postoperatively, both before and after matching.

Attribute	Before matching						After matching					
	pH > 7.3		pH ≤ 7.3				pH > 7.3		pH ≤ 7.3			
Patient numbers	N = 702		N = 29				N = 27		N = 27			
Demographics	N	%	N	%	p-value	SMD	N	%	N	%	p-value	SMD
Age												
Current Age	71.8 ± 13.6		70.1 ± 17.2		0.501	0.113	71 ± 11.1		70.6 ± 17.5		0.926	0.025
Age at Index	64.2 ± 13.5		62.9 ± 15.7		0.615	0.088	62.8 ± 11.1		63.4 ± 16.2		0.876	0.043
Sex												
Male	402	57.3%	17	58.6%	0.885	0.027	19	70.4%	16	59.3%	0.393	0.234
Female	284	40.5%	12	41.4%	0.921	0.019	≤10	37.0%	11	40.7%	0.780	0.076
Ethnicity												
Not Hispanic or Latino	491	69.9%	16	55.2%	0.091	0.309	12	44.4%	16	59.3%	0.276	0.300
Hispanic or Latino	62	8.8%	≤10	34.5%	<0.001	0.655	≤10	37.0%	≤10	37.0%	1.000	<0.001
Unknown Ethnicity	149	21.2%	≤10	34.5%	0.090	0.299	≤10	37.0%	≤10	37.0%	1.000	<0.001
Race												
White	494	70.4%	19	65.5%	0.576	0.104	12	44.4%	17	63.0%	0.172	0.378
Asian	15	2.1%	≤10	34.5%	<0.001	0.921	≤10	37.0%	≤10	37.0%	1.000	<0.001
Black or African American	93	13.2%	≤10	34.5%	0.001	0.514	≤10	37.0%	≤10	37.0%	1.000	<0.001
American Indian or Alaska Native	≤10	1.4%	0	0.0%	0.518	0.170	0	0.0%	0	0.0%	--	--
Native Hawaiian or Other Pacific Islander	0	0.0%	0	0.0%	--	--	0	0.0%	0	0.0%	--	--
Other Race	14	2.0%	≤10	34.5%	<0.001	0.927	≤10	37.0%	≤10	37.0%	1.000	<0.001
Unknown Race	85	12.1%	≤10	34.5%	<0.001	0.549	≤10	37.0%	≤10	37.0%	1.000	<0.001
Diagnosis												
Hypertensive diseases	556	79.2%	24	82.8%	0.643	0.091	22	81.5%	22	81.5%	1.000	<0.001
Diabetes mellitus	281	40.0%	18	62.1%	0.018	0.452	17	63.0%	17	63.0%	1.000	<0.001
Disorders of lipoprotein metabolism and other lipidemias	366	52.1%	16	55.2%	0.748	0.061	13	48.1%	15	55.6%	0.586	0.149

Cerebrovascular diseases	100	14.2%	≤10	34.5%	0.003	0.485	≤10	37.0%	≤10	37.0%	1.000	<0.001
Heart failure	116	16.5%	≤10	34.5%	0.012	0.421	≤10	37.0%	≤10	37.0%	1.000	<0.001
Ischemic heart diseases	209	29.8%	15	51.7%	0.012	0.458	12	44.4%	13	48.1%	0.785	0.074
Nicotine dependence, unspecified, uncomplicated	77	11.0%	≤10	34.5%	<0.001	0.585	≤10	37.0%	≤10	37.0%	1.000	<0.001
BMI	30.4 ± 7.9		32.3 ± 7.8		0.256	0.243	30.1 ± 6.8		31.5 ± 7.3		0.509	0.203
0 - 18.50 kg/m2	37	5.3%	≤10	34.5%	<0.001	0.787	≤10	37.0%	≤10	37.0%	1.000	<0.001
18.50 - 25 kg/m2	177	25.2%	≤10	34.5%	0.262	0.204	≤10	37.0%	≤10	37.0%	1.000	<0.001
25 - 30 kg/m2	284	40.5%	13	44.8%	0.639	0.088	15	55.6%	13	48.1%	0.586	0.149
30 - 0 kg/m2	321	45.7%	19	65.5%	0.036	0.406	13	48.1%	17	63.0%	0.273	0.302
Glomerular filtration rate	34.7 ± 18.0		40.1 ± 19.9		0.122	0.285	32.0 ± 10.2		39.3 ± 19.8		0.107	0.463
15 - 20 ml/min/1.73m^2	202	28.8%	≤10	34.5%	0.507	0.123	≤10	37.0%	≤10	37.0%	1.000	<0.001
20 - 25 ml/min/1.73m^2	354	50.4%	13	44.8%	0.554	0.112	12	44.4%	13	48.1%	0.785	0.074
25 - 30 ml/min/1.73m^2	500	71.2%	20	69.0%	0.792	0.049	20	74.1%	19	70.4%	0.761	0.083

[1]To safeguard patients' Protected health information (PHI), 1 to 9 cases are always rounded up to 10.

Supplementary Table 3. Demographic and clinical characteristics of patients with blood $HCO3^-$ > 22 and $HCO3^-$ ≤ 22 (mmol/L), assessed between 1 week and 1 month postoperatively, both before and after matching.

Attribute	Before matching						After matching					
	$HCO3^-$ > 22		$HCO3^-$ ≤ 22				$HCO3^-$ > 22		$HCO3^-$ ≤ 22			
Patient numbers	N = 443		N = 288				N = 256		N = 256			
Demographics	N	%	N	%	p-value	SMD	N	%	N	%	p-value	SMD
Age												
Current Age	72.3 ± 13.0		70.9 ± 14.9		0.162	0.105	71.0 ± 13.2		71.2 ± 14.5		0.896	0.012
Age at Index	64.3 ± 12.8		63.8 ± 14.7		0.637	0.035	63.7 ± 12.9		63.9 ± 14.4		0.907	0.010
Sex												
Male	232	52.4%	187	64.9%	0.001	0.257	163	63.7%	160	62.5%	0.784	0.024
Female	200	45.1%	96	33.3%	0.001	0.244	91	35.5%	91	35.5%	1.000	<0.001
Ethnicity												
Not Hispanic or Latino	311	70.2%	196	68.1%	0.538	0.047	181	70.7%	175	68.4%	0.565	0.051
Hispanic or Latino	39	8.8%	28	9.7%	0.674	0.032	25	9.8%	23	9.0%	0.762	0.027
Unknown Ethnicity	93	21.0%	64	22.2%	0.693	0.030	50	19.5%	58	22.7%	0.386	0.077
Race												
White	315	71.1%	198	68.8%	0.496	0.051	185	72.3%	180	70.3%	0.625	0.043
Asian	≤10	2.3%	≤10	3.5%	0.325	0.073	≤10	3.9%	≤10	3.9%	1.000	<0.001
Black or African American	61	13.8%	36	12.5%	0.621	0.038	37	14.5%	34	13.3%	0.701	0.034
American Indian or Alaska Native	≤10	2.3%	0	0.0%	0.010	0.215	0	0.0%	0	0.0%	--	--
Native Hawaiian or Other Pacific Islander	0	.0.0%	0	0.0%	--	--	0	0.0%	0	0.0%	--	--
Other Race	≤10	2.3%	≤10	3.5%	0.325	0.073	≤10	3.9%	≤10	3.9%	1.000	<0.001
Unknown Race	54	12.2%	35	12.2%	0.988	0.001	25	9.8%	31	12.1%	0.396	0.075
Diagnosis												
Hypertensive diseases	357	80.6%	223	77.4%	0.303	0.078	204	79.7%	201	78.5%	0.744	0.029
Diabetes mellitus	185	41.8%	114	39.6%	0.558	0.044	105	41.0%	101	39.5%	0.718	0.032
Disorders of lipoprotein metabolism and other lipidemias	235	53.0%	147	51.0%	0.596	0.040	139	54.3%	133	52.0%	0.595	0.047

Cerebrovascular diseases	62	14.0%	42	14.6%	0.824	0.017	37	14.5%	40	15.6%	0.711	0.033
Heart failure	71	16.0%	53	18.4%	0.403	0.063	41	16.0%	43	16.8%	0.811	0.021
Ischemic heart diseases	124	28.0%	100	34.7%	0.054	0.145	79	30.9%	81	31.6%	0.849	0.017
Nicotine dependence, unspecified, uncomplicated	49	11.1%	30	10.4%	0.784	0.021	29	11.3%	25	9.8%	0.565	0.051
BMI	31.1 ± 8.3		29.5 ± 7.0		0.016	0.208	30.9 ± 8.4		29.9 ± 7.1		0.208	0.125
0 - 18.50 kg/m2	24	5.4%	15	5.2%	0.902	0.009	17	6.6%	14	5.5%	0.578	0.049
18.50 - 25 kg/m2	98	22.1%	85	29.5%	0.024	0.170	67	26.2%	64	25.0%	0.761	0.027
25 - 30 kg/m2	178	40.2%	119	41.3%	0.759	0.023	105	41.0%	105	41.0%	1.000	<0.001
30 - 0 kg/m2	209	47.2%	131	45.5%	0.654	0.034	119	46.5%	118	46.1%	0.929	0.008
Glomerular filtration rate	35.5 ± 18.2		34.0 ± 17.9		0.297	0.081	34.8 ± 17.6		34.4 ± 18.1		0.779	0.025
15 - 20 ml/min/1.73m^2	121	27.3%	89	30.9%	0.295	0.079	82	32.0%	76	29.7%	0.566	0.051
20 - 25 ml/min/1.73m^2	222	50.1%	145	50.3%	0.951	0.005	134	52.3%	131	51.2%	0.791	0.023
25 - 30 ml/min/1.73m^2	321	72.5%	199	69.1%	0.327	0.074	174	68.0%	182	71.1%	0.442	0.068

[1]To safeguard patients' Protected health information (PHI), 1 to 9 cases are always rounded up to 10.

Supplementary Table 4. Demographic and clinical characteristics of patients with blood Na^+ wnl and Na^+ abn, assessed between 1 week and 1 month postoperatively, both before and after matching.

Attribute	Before matching						After matching					
	Na^+ wnl		Na^+ abn				Na^+ wnl		Na^+ abn			
Patient numbers	N = 448		N = 283				N = 250		N = 250			
Demographics	N	%	N	%	p-value	SMD	N	%	N	%	p-value	SMD
Age												
Current Age	72.3 ± 12.9		70.9 ± 15.1		0.192	0.097	72.1 ± 13.9		71.4 ± 14.7		0.553	0.053
Age at Index	64.4 ± 12.7		63.7 ± 15.0		0.448	0.057	64.8 ± 13.4		64.1 ± 14.7		0.557	0.053
Sex												
Male	240	53.6%	179	63.3%	0.010	0.197	149	59.6%	151	60.4%	0.855	0.016
Female	199	44.4%	97	34.3%	0.006	0.209	95	38.0%	93	37.2%	0.854	0.017
Ethnicity												
Not Hispanic or Latino	316	70.5%	191	67.5%	0.384	0.066	175	7.0%	172	68.8%	0.771	0.026
Hispanic or Latino	41	9.2%	26	9.2%	0.987	0.001	27	10.8%	22	8.8%	0.452	0.067
Unknown Ethnicity	91	20.3%	66	23.3%	0.335	0.073	48	19.2%	56	22.4%	0.378	0.079
Race												
White	313	69.9%	200	70.7%	0.817	0.018	181	72.4%	178	71.2%	0.766	0.027
Asian	11	2.5%	≤10	3.5%	0.395	0.063	≤10	4.0%	≤10	4.0%	1.000	<0.001
Black or African American	65	14.5%	32	11.3%	0.214	0.096	29	11.6%	31	12.4%	0.783	0.025
American Indian or Alaska Native	0	0.0%	≤10	3.5%	<0.001	0.271	0	0.0%	0	0.0%	--	--
Native Hawaiian or Other Pacific Islander	0	0.0%	0	0.0%	--	--	0	0.0%	0	0.0%	--	--
Other Race	≤10	2.2%	≤10	3.5%	0.293	0.078	≤10	4.0%	≤10	4%	1.000	<0.001
Unknown Race	51	11.4%	38	13.4%	0.410	0.062	29	11.6%	32	12.8%	0.682	0.037
Diagnosis												
Hypertensive diseases	356	79.5%	224	79.2%	0.919	0.008	199	79.6%	199	79.6%	1.000	<0.001
Diabetes mellitus	184	41.1%	115	40.6%	0.907	0.009	95	38.0%	102	40.8%	0.522	0.057
Disorders of lipoprotein metabolism and other lipidemias	237	52.9%	145	51.2%	0.661	0.033	133	53.2%	131	52.4%	0.858	0.016

Cerebrovascular diseases	60	13.4%	44	15.5%	0.417	0.061	40	16.0%	37	14.8%	0.710	0.033
Heart failure	60	13.4%	64	22.6%	0.001	0.242	41	16.4%	41	16.4%	1.000	<0.001
Ischemic heart diseases	140	31.3%	84	29.7%	0.654	0.034	76	30.4%	72	28.8%	0.695	0.035
Nicotine dependence, unspecified, uncomplicated	46	10.3%	33	11.7%	0.555	0.045	28	11.2%	26	10.4%	0.773	0.026
BMI	30.9 ± 7.9		29.8 ± 7.8		0.098	0.141	29.8 ± 7.7		29.9 ± 7.9		0.878	0.015
0 - 18.50 kg/m2	25	5.6%	14	4.9%	0.711	0.028	16	6.4%	14	5.6%	0.706	0.034
18.50 - 25 kg/m2	103	23.0%	80	28.3%	0.109	0.121	71	28.4%	64	25.6%	0.481	0.063
25 - 30 kg/m2	179	40.0%	118	41.7%	0.641	0.035	114	45.6%	103	41.2%	0.321	0.089
30 - 0 kg/m2	219	48.9%	121	42.8%	0.106	0.123	108	43.2%	111	44.4%	0.787	0.024
Glomerular filtration rate	34.1 ± 15.0		36.2 ± 22.1		0.144	0.109	34.9 ± 15.1		36.4 ± 22.5		0.394	0.079
15 - 20 ml/min/1.73m^2	128	28.6%	82	29.0%	0.906	0.009	71	28.4%	72	28.8%	0.921	0.009
20 - 25 ml/min/1.73m^2	230	51.3%	137	48.4%	0.440	0.059	123	49.2%	123	49.2%	1.000	<0.001
25 - 30 ml/min/1.73m^2	329	73.4%	191	67.5%	0.084	0.131	169	67.6%	173	69.2%	0.700	0.034

[1]To safeguard patients' Protected health information (PHI), 1 to 9 cases are always rounded up to 10.

Supplementary Table 5. Demographic and clinical characteristics of patients with blood K^+ wnl and K^+ abn, assessed between 1 week and 1 month postoperatively, both before and after matching.

Attribute	Before matching						After matching					
	K^+ wnl		K^+ abn				K^+ wnl		K^+ abn			
Patient numbers	N = 522		N = 209				N = 199		N = 199			
Demographics	N	%	N	%	p-value	SMD	N	%	N	%	p-value	SMD
Age												
Current Age	71.6 ± 13.1		72.1 ± 15.4		0.700	0.03	70.5 ± 13.9		72.1 ± 15.4		0.287	0.107
Age at Index	64.0 ± 13.0		64.6 ± 15.1		0.600	0.041	63.2 ± 13.9		64.5 ± 15.2		0.354	0.093
Sex												
Male	290	55.6%	129	61.7%	0.128	0.125	116	58.3%	121	60.8%	0.610	0.051
Female	218	41.8%	78	37.3%	0.269	0.091	81	40.7%	76	38.2%	0.608	0.051
Ethnicity												
Not Hispanic or Latino	363	69.5%	144	68.9%	0.865	0.014	142	71.4%	139	69.8%	0.741	0.033
Hispanic or Latino	50	9.6%	17	8.1%	0.541	0.051	16	8.0%	16	8.0%	1.000	<0.001
Unknown Ethnicity	109	20.9%	48	23.0%	0.535	0.050	41	20.6%	44	22.1%	0.714	0.037
Race												
White	367	70.3%	146	69.9%	0.904	0.010	140	70.4%	140	70.4%	1.000	<0.001
Asian	≤10	1.9%	≤10	4.8%	0.032	0.160	≤10	5.0%	≤10	5.0%	1.000	<0.001
Black or African American	71	13.6%	26	12.4%	0.676	0.035	29	14.6%	26	13.1%	0.663	0.044
American Indian or Alaska Native	≤10	1.9%	0	0.0%	0.044	0.198	0	0.0%	0	0.0%	--	--
Native Hawaiian or Other Pacific Islander	0	0.0%	0	0.0%	--	--	0	0.0%	0	0.0%	--	--
Other Race	11	2.1%	≤10	4.8%	0.050	0.147	≤10	5.0%	≤10	5.0%	1.000	<0.001
Unknown Race	63	12.1%	26	12.4%	0.890	0.011	23	11.6%	25	12.6%	0.758	0.031
Diagnosis												
Hypertensive diseases	412	78.9%	168	80.4%	0.660	0.036	154	77.4%	159	79.9%	0.541	0.061
Diabetes mellitus	213	40.8%	86	41.1%	0.932	0.007	78	39.2%	81	40.7%	0.759	0.031
Disorders of lipoprotein metabolism and other lipidemias	276	52.9%	106	50.7%	0.598	0.043	99	49.7%	103	51.8%	0.688	0.040

Cerebrovascular diseases	70	13.4%	34	16.3%	0.318	0.080	35	17.6%	31	15.6%	0.590	0.054
Heart failure	84	16.1%	40	19.1%	0.321	0.080	31	15.6%	38	19.1%	0.354	0.093
Ischemic heart diseases	155	29.7%	69	33.0%	0.379	0.072	59	29.6%	65	32.7%	0.516	0.065
Nicotine dependence, unspecified, uncomplicated	53	10.2%	26	12.4%	0.368	0.072	26	13.1%	24	12.1%	0.762	0.030
BMI	30.6 ± 7.6		30.3 ± 8.4		0.761	0.027	30.0 ± 7.2		30.4 ± 8.4		0.640	0.051
0 - 18.50 kg/m2	29	5.6%	≤10	4.8%	0.675	0.035	≤10	5.0%	≤10	5.0%	1.000	<0.001
18.50 - 25 kg/m2	123	23.6%	60	28.7%	0.147	0.117	60	30.2%	57	28.6%	0.741	0.033
25 - 30 kg/m2	203	38.9%	94	45.0%	0.130	0.124	92	46.2%	88	44.2%	0.687	0.040
30 - 0 kg/m2	233	44.6%	107	51.2%	0.108	0.132	101	50.8%	99	49.7%	0.841	0.020
Glomerular filtration rate	34.3 ± 17.0		36.3 ± 20.5		0.186	0.106	33.5 ± 19.5		35.6 ± 19.4		0.290	0.108
15 - 20 ml/min/1.73m2	148	28.4%	62	29.7%	0.723	0.029	61	30.7%	59	29.6%	0.827	0.022
20 - 25 ml/min/1.73m2	269	51.5%	98	46.9%	0.257	0.093	100	50.3%	94	47.2%	0.547	0.060
25 - 30 ml/min/1.73m2	374	71.6%	146	69.9%	0.629	0.039	134	67.3%	140	70.4%	0.516	0.065

[1]To safeguard patients' Protected health information (PHI), 1 to 9 cases are always rounded up to 10.

Supplementary Table 6. Demographic and clinical characteristics of patients with blood Ca^{2+} wnl and Ca^{2+} abn, assessed between 1 week and 1 month postoperatively, both before and after matching.

Attribute	Before matching						After matching					
	Ca^{2+} wnl		Ca^{2+} abn				Ca^{2+} wnl		Ca^{2+} abn			
Patient numbers	N = 367		N = 364				N = 286		N = 286			
Demographics	N	%	N	%	p-value	SMD	N	%	N	%	p-value	SMD
Age												
Current Age	71.9 ± 12.7		71.7 ± 14.8		0.860	0.013	72.1 ± 12.7		71.7 ± 14.4		0.738	0.028
Age at Index	63.9 ± 12.7		64.3 ± 14.5		0.695	0.029	64.4 ± 12.4		63.9 ± 13.9		0.657	0.037
Sex												
Male	186	50.7%	233	64.0%	<0.001	0.272	164	57.3%	168	58.7%	0.735	0.028
Female	172	46.9%	124	34.1%	<0.001	0.263	117	40.9%	111	38.8%	0.608	0.043
Ethnicity												
Not Hispanic or Latino	253	68.9%	254	69.8%	0.805	0.018	202	70.6%	191	66.8%	0.321	0.083
Hispanic or Latino	39	10.6%	28	7.7%	0.169	0.102	22	7.7%	26	9.1%	0.546	0.050
Unknown Ethnicity	75	20.4%	82	22.5%	0.491	0.051	62	21.7%	69	24.1%	0.486	0.058
Race												
White	261	71.1%	252	69.2%	0.577	0.041	210	73.4%	198	69.2%	0.267	0.093
Asian	≤10	2.7%	≤10	2.7%	0.985	0.001	≤10	3.5%	≤10	3.5%	1.000	<0.001
Black or African American	51	13.9%	46	12.6%	0.616	0.037	37	12.9%	37	12.9%	1.000	<0.001
American Indian or Alaska Native	0	0.0%	≤10	2.7%	0.001	0.238	0	0.0%	0	0.0%	--	--
Native Hawaiian or Other Pacific Islander	0	0.0%	0	0.0%	--	--	0	0.0%	0	0.0%	--	--
Other Race	≤10	2.7%	≤10	2.7%	0.985	0.001	≤10	3.5%	≤10	3.5%	1.000	<0.001
Unknown Race	43	11.7%	46	12.6%	0.703	0.028	27	9.4%	40	14.0%	0.091	0.142
Diagnosis												
Hypertensive diseases	299	81.5%	281	77.2%	0.154	0.106	228	79.7%	229	80.1%	0.917	0.009
Diabetes mellitus	152	41.4%	147	40.4%	0.777	0.021	117	40.9%	120	42.0%	0.799	0.021
Disorders of lipoprotein metabolism and other lipidemias	200	54.5%	182	5.0%	0.224	0.090	150	52.4%	150	52.4%	1.000	<0.001

Cerebrovascular diseases	47	12.8%	57	15.7%	0.270	0.082	40	14.0%	41	14.3%	0.905	0.010
Heart failure	49	13.4%	75	20.6%	0.009	0.194	49	17.1%	47	16.4%	0.823	0.019
Ischemic heart diseases	100	27.2%	124	34.1%	0.046	0.148	94	32.9%	89	31.1%	0.654	0.037
Nicotine dependence, unspecified, uncomplicated	45	12.3%	34	9.3%	0.203	0.094	28	9.8%	27	9.4%	0.887	0.012
BMI	30.9 ± 8.3		30.0 ± 7.4		0.172	0.113	30.3 ± 7.4		30.4 ± 7.5		0.793	0.025
0 - 18.50 kg/m2	19	5.2%	20	5.5%	0.849	0.014	16	5.6%	17	5.9%	0.858	0.015
18.50 - 25 kg/m2	88	24.0%	95	26.1%	0.508	0.049	73	25.5%	71	24.8%	0.847	0.016
25 - 30 kg/m2	149	40.6%	148	40.7%	0.987	0.001	121	42.3%	115	40.2%	0.610	0.043
30 - 0 kg/m2	176	48.0%	164	45.1%	0.432	0.058	130	45.5%	138	48.3%	0.503	0.056
Glomerular filtration rate	33.7 ± 15.3		36.1 ± 20.5		0.082	0.132	33.1 ± 14.8		36.8 ± 21.6		0.021	0.199
15 - 20 ml/min/1.73m^2	106	28.9%	104	28.6%	0.926	0.007	81	28.3%	82	28.7%	0.926	0.008
20 - 25 ml/min/1.73m^2	193	52.6%	174	47.8%	0.196	0.096	143	5.0%	141	49.3%	0.867	0.014
25 - 30 ml/min/1.73m^2	272	74.1%	248	68.1%	0.074	0.132	205	71.7%	203	71.0%	0.853	0.015

[1]To safeguard patients' Protected health information (PHI), 1 to 9 cases are always rounded up to 10.

Supplementary Table 7. Demographic and clinical characteristics of patients with blood Mg^{2+} wnl and Mg^{2+} abn, assessed between 1 week and 1 month postoperatively, both before and after matching.

Attribute	Before matching						After matching					
	Mg^{2+} wnl		Mg^{2+} abn				Mg^{2+} wnl		Mg^{2+} abn			
Patient numbers	N = 535		N = 196				N = 184		N = 184			
Demographics	N	%	N	%	p-value	SMD	N	%	N	%	p-value	SMD
Age												
Current Age	72.1 ± 13.0		70.7 ± 15.7		0.224	0.097	70.5 ± 14.3		71.2 ± 15.4		0.659	0.046
Age at Index	64.4 ± 12.9		63.3 ± 15.4		0.336	0.077	63.5 ± 14.1		63.9 ± 15.3		0.777	0.03
Sex												
Male	300	56.1%	119	60.7%	0.261	0.094	109	59.2%	112	60.9%	0.750	0.033
Female	221	41.3%	75	38.3%	0.458	0.062	73	39.7%	70	38.0%	0.748	0.033
Ethnicity												
Not Hispanic or Latino	378	70.7%	129	65.8%	0.209	0.104	121	65.8%	128	69.6%	0.435	0.081
Hispanic or Latino	49	9.2%	18	9.2%	0.992	0.001	20	10.9%	16	8.7%	0.483	0.073
Unknown Ethnicity	108	20.2%	49	25%	0.160	0.115	43	23.4%	40	21.7%	0.708	0.039
Race												
White	385	72.0%	128	65.3%	0.081	0.144	117	63.6%	125	67.9%	0.379	0.092
Asian	≤10	1.9%	≤10	5.1%	0.018	0.177	≤10	5.4%	≤10	5.4%	1.000	<0.001
Black or African American	71	13.3%	26	13.3%	0.998	<0.001	29	15.8%	25	13.6%	0.556	0.061
American Indian or Alaska Native	≤10	1.9%	0	0.0%	0.054	0.195	0	0.0%	0	0.0%	--	--
Native Hawaiian or Other Pacific Islander	0	0.0%	0	0.0%	--	--	0	0.0%	0	0.0%	--	--
Other Race	≤10	1.9%	≤10	5.1%	0.018	0.177	≤10	5.4%	≤10	5.4%	1.000	<0.001
Unknown Race	59	11.0%	30	15.3%	0.117	0.127	25	13.6%	24	13.0%	0.878	0.016
Diagnosis												
Hypertensive diseases	428	8.0%	152	77.6%	0.469	0.06	150	81.5%	144	78.3%	0.435	0.081
Diabetes mellitus	215	40.2%	84	42.9%	0.515	0.054	86	46.7%	78	42.4%	0.401	0.088
Disorders of lipoprotein metabolism and other lipidemias	285	53.3%	97	49.5%	0.365	0.076	100	54.3%	93	50.5%	0.465	0.076

Cerebrovascular diseases	68	12.7%	36	18.4%	0.052	0.157	35	19.0%	30	16.3%	0.494	0.071
Heart failure	77	14.4%	47	24.0%	0.002	0.245	43	23.4%	39	21.2%	0.616	0.052
Ischemic heart diseases	153	28.6%	71	36.2%	0.048	0.163	69	37.5%	66	35.9%	0.746	0.034
Nicotine dependence, unspecified, uncomplicated	59	11.0%	20	10.2%	0.751	0.027	24	13.0%	19	10.3%	0.417	0.085
BMI	30.8 ± 7.8		29.8 ± 7.9		0.175	0.125	30.4 ± 7.6		30.0 ± 8.0		0.656	0.052
0 - 18.50 kg/m2	24	4.5%	15	7.7%	0.091	0.133	13	7.1%	13	7.1%	1.000	<0.001
18.50 - 25 kg/m2	121	22.6%	62	31.6%	0.013	0.204	50	27.2%	55	29.9%	0.564	0.060
25 - 30 kg/m2	220	41.1%	77	39.3%	0.654	0.037	66	35.9%	73	39.7%	0.452	0.079
30 - 0 kg/m2	250	46.7%	90	45.9%	0.846	0.016	85	46.2%	86	46.7%	0.917	0.011
Glomerular filtration rate	33.7 ± 15.8		38.3 ± 22.9		0.003	0.236	33.3 ± 16.8		38.2 ± 22.7		0.023	0.243
15 - 20 ml/min/1.73m^2	156	29.2%	54	27.6%	0.670	0.036	57	31.0%	49	26.6%	0.357	0.096
20 - 25 ml/min/1.73m^2	280	52.3%	87	44.4%	0.057	0.160	94	51.1%	81	44.0%	0.175	0.142
25 - 30 ml/min/1.73m^2	380	71.0%	140	71.4%	0.916	0.009	135	73.4%	129	70.1%	0.487	0.072

[1]To safeguard patients' Protected health information (PHI), 1 to 9 cases are always rounded up to 10.

Supplementary Table 8. Demographic and clinical characteristics of patients with blood P wnl and P abn, assessed between 1 week and 1 month postoperatively, both before and after matching.

Attribute	Before matching						After matching					
	P wnl		P abn				P wnl		P abn			
Patient numbers	N = 524		N = 207				N = 202		N = 202			
Demographics	N	%	N	%	p-value	SMD	N	%	N	%	p-value	SMD
Age												
Current Age	72.2 ± 12.9		70.6 ± 15.7		0.154	0.112	70.5 ± 14.1		70.6 ± 15.8		0.931	0.009
Age at Index	64.5 ± 12.8		63.2 ± 15.5		0.256	0.089	62.7 ± 14.1		63.2 ± 15.6		0.710	0.037
Sex												
Male	286	54.6%	133	64.3%	0.017	0.198	134	66.3%	130	64.4%	0.676	0.042
Female	224	42.7%	72	34.8%	0.048	0.164	65	32.2%	70	34.7%	0.598	0.052
Ethnicity												
Not Hispanic or Latino	369	70.4%	138	66.7%	0.321	0.081	137	67.8%	135	66.8%	0.832	0.021
Hispanic or Latino	50	9.5%	17	8.2%	0.575	0.047	14	6.9%	17	8.4%	0.575	0.056
Unknown Ethnicity	105	20.0%	52	25.1%	0.132	0.122	51	25.2%	50	24.8%	0.909	0.011
Race												
White	371	70.8%	142	68.6%	0.558	0.048	142	70.3%	141	69.8%	0.914	0.011
Asian	≤10	1.9%	≤10	4.8%	0.029	0.162	≤10	5.0%	≤10	5.0%	1.000	<0.001
Black or African American	74	14.1%	23	11.1%	0.280	0.091	22	10.9%	23	11.4%	0.874	0.016
American Indian or Alaska Native	≤10	1.9%	0	0.0%	0.045	0.197	0	0.0%	0	0.0%	--	--
Native Hawaiian or Other Pacific Islander	0	0.0%	0	0.0%	--	--	0	0.0%	0	0.0%	--	--
Other Race	≤10	1.9%	≤10	4.8%	0.029	0.162	≤10	5.0%	≤10	5.0%	1.000	<0.001
Unknown Race	60	11.5%	29	14.0%	0.340	0.077	27	13.4%	28	13.9%	0.885	0.014
Diagnosis												
Hypertensive diseases	419	80.0%	161	77.8%	0.511	0.054	160	79.2%	157	77.7%	0.717	0.036
Diabetes mellitus	213	40.6%	86	41.5%	0.824	0.018	76	37.6%	84	41.6%	0.416	0.081
Disorders of lipoprotein metabolism and other lipidemias	277	52.9%	105	50.7%	0.602	0.043	96	47.5%	102	50.5%	0.550	0.059

Cerebrovascular diseases	73	13.9%	31	15.0%	0.716	0.030	21	10.4%	30	14.9%	0.178	0.134
Heart failure	80	15.3%	44	21.3%	0.052	0.155	36	17.8%	42	20.8%	0.449	0.075
Ischemic heart diseases	154	29.4%	70	33.8%	0.242	0.095	66	32.7%	67	33.2%	0.916	0.011
Nicotine dependence, unspecified, uncomplicated	60	11.5%	19	9.2%	0.373	0.075	15	7.4%	19	9.4%	0.473	0.071
BMI	30.8 ± 8.0		29.7 ± 7.5		0.138	0.137	30.0 ± 8.2		29.8 ± 7.4		0.788	0.030
0 - 18.50 kg/m2	27	5.2%	12	5.8%	0.727	0.028	≤10	5.0%	11	5.4%	0.823	0.022
18.50 - 25 kg/m2	123	23.5%	60	29.0%	0.121	0.126	62	30.7%	56	27.7%	0.512	0.065
25 - 30 kg/m2	215	41.0%	82	39.6%	0.725	0.029	84	41.6%	80	39.6%	0.685	0.040
30 - 0 kg/m2	246	46.9%	94	45.4%	0.708	0.031	89	44.1%	93	46.0%	0.689	0.040
Glomerular filtration rate	34.0 ± 15.8		37.2 ± 22.8		0.035	0.163	33.6 ± 16.2		37.1 ± 22.8		0.084	0.177
15 - 20 ml/min/1.73m^2	152	29.0%	58	28.0%	0.790	0.022	58	28.7%	57	28.2%	0.912	0.011
20 - 25 ml/min/1.73m^2	271	51.7%	96	46.4%	0.193	0.107	97	48.0%	96	47.5%	0.921	0.010
25 - 30 ml/min/1.73m^2	379	72.3%	141	68.1%	0.257	0.092	134	66.3%	139	68.8%	0.595	0.053

[1]To safeguard patients' Protected health information (PHI), 1 to 9 cases are always rounded up to 10.

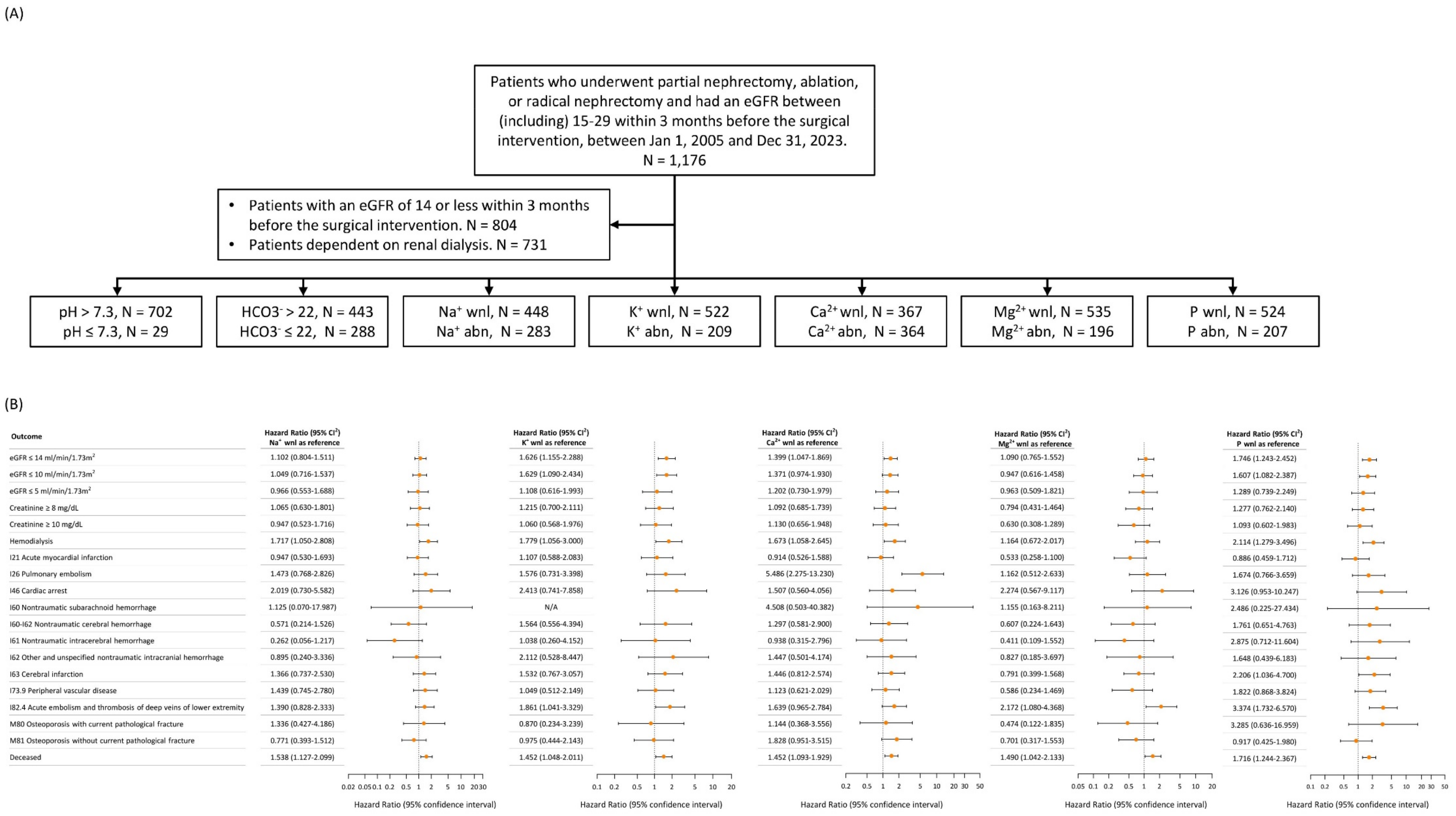

Outcome	Hazard Ratio (95% CI[2]) Na⁺ wnl as reference	Hazard Ratio (95% CI[2]) K⁺ wnl as reference	Hazard Ratio (95% CI[2]) Ca²⁺ wnl as reference	Hazard Ratio (95% CI[2]) Mg²⁺ wnl as reference	Hazard Ratio (95% CI[2]) P wnl as reference
eGFR ≤ 14 ml/min/1.73m²	1.102 (0.804-1.511)	1.626 (1.155-2.288)	1.399 (1.047-1.869)	1.090 (0.765-1.552)	1.746 (1.243-2.452)
eGFR ≤ 10 ml/min/1.73m²	1.049 (0.716-1.537)	1.629 (1.090-2.434)	1.371 (0.974-1.930)	0.947 (0.616-1.458)	1.607 (1.082-2.387)
eGFR ≤ 5 ml/min/1.73m²	0.966 (0.553-1.688)	1.108 (0.616-1.993)	1.202 (0.730-1.979)	0.963 (0.509-1.821)	1.289 (0.739-2.249)
Creatinine ≥ 8 mg/dL	1.065 (0.630-1.801)	1.215 (0.700-2.111)	1.092 (0.685-1.739)	0.794 (0.431-1.464)	1.277 (0.762-2.140)
Creatinine ≥ 10 mg/dL	0.947 (0.523-1.716)	1.060 (0.568-1.976)	1.130 (0.656-1.948)	0.630 (0.308-1.289)	1.093 (0.602-1.983)
Hemodialysis	1.717 (1.050-2.808)	1.779 (1.056-3.000)	1.673 (1.058-2.645)	1.164 (0.672-2.017)	2.114 (1.279-3.496)
I21 Acute myocardial infarction	0.947 (0.530-1.693)	1.107 (0.588-2.083)	0.914 (0.526-1.588)	0.533 (0.258-1.100)	0.886 (0.459-1.712)
I26 Pulmonary embolism	1.473 (0.768-2.826)	1.576 (0.731-3.398)	5.486 (2.275-13.230)	1.162 (0.512-2.633)	1.674 (0.766-3.659)
I46 Cardiac arrest	2.019 (0.730-5.582)	2.413 (0.741-7.858)	1.507 (0.560-4.056)	2.274 (0.567-9.117)	3.126 (0.953-10.247)
I60 Nontraumatic subarachnoid hemorrhage	1.125 (0.070-17.987)	N/A	4.508 (0.503-40.382)	1.155 (0.163-8.211)	2.486 (0.225-27.434)
I60-I62 Nontraumatic cerebral hemorrhage	0.571 (0.214-1.526)	1.564 (0.556-4.394)	1.297 (0.581-2.900)	0.607 (0.224-1.643)	1.761 (0.651-4.763)
I61 Nontraumatic intracerebral hemorrhage	0.262 (0.056-1.217)	1.038 (0.260-4.152)	0.938 (0.315-2.796)	0.411 (0.109-1.552)	2.875 (0.712-11.604)
I62 Other and unspecified nontraumatic intracranial hemorrhage	0.895 (0.240-3.336)	2.112 (0.528-8.447)	1.447 (0.501-4.174)	0.827 (0.185-3.697)	1.648 (0.439-6.183)
I63 Cerebral infarction	1.366 (0.737-2.530)	1.532 (0.767-3.057)	1.446 (0.812-2.574)	0.791 (0.399-1.568)	2.206 (1.036-4.700)
I73.9 Peripheral vascular disease	1.439 (0.745-2.780)	1.049 (0.512-2.149)	1.123 (0.621-2.029)	0.586 (0.234-1.469)	1.822 (0.868-3.824)
I82.4 Acute embolism and thrombosis of deep veins of lower extremity	1.390 (0.828-2.333)	1.861 (1.041-3.329)	1.639 (0.965-2.784)	2.172 (1.080-4.368)	3.374 (1.732-6.570)
M80 Osteoporosis with current pathological fracture	1.336 (0.427-4.186)	0.870 (0.234-3.239)	1.144 (0.368-3.556)	0.474 (0.122-1.835)	3.285 (0.636-16.959)
M81 Osteoporosis without current pathological fracture	0.771 (0.393-1.512)	0.975 (0.444-2.143)	1.828 (0.951-3.515)	0.701 (0.317-1.553)	0.917 (0.425-1.980)
Deceased	1.538 (1.127-2.099)	1.452 (1.048-2.011)	1.452 (1.093-1.929)	1.490 (1.042-2.133)	1.716 (1.244-2.367)

Supplementary Figure 1. Comparison of renal function and long-term outcomes between patients with normal and abnormal electrolyte parameters measured between 1 week and 1 month, regardless of surgical intervention type.

Outcome	Patients with outcome pH > 7.3 / ≤ 7.3	Median survival[1] (days) pH > 7.3 / ≤ 7.3	Survival probability[1] (%) pH > 7.3 / ≤ 7.3	Hazard Ratio (95% CI[2]) pH > 7.3 as reference
eGFR ≤ 14 ml/min/1.73m^2	12/≤10	2277/NR	36.04/72.46	0.488 (0.171-1.388)
eGFR ≤ 10 ml/min/1.73m^2	11/≤10	2365/NR	39.24/68.73	0.517 (0.163-1.638)
eGFR ≤ 5 ml/min/1.73m^2	≤10/0	NR/NR	53.17/100.00	N/A
Creatinine ≥ 8 mg/dL	≤10/≤10	NR/NR	52.41/87.50	0.363 (0.077-1.713)
Creatinine ≥ 10 mg/dL	≤10/0	3079/NR	48.46/100.00	N/A
Hemodialysis	≤10/≤10	NR/NR	57.02/87.13	0.573 (0.147-2.231)
I21 Acute myocardial infarction	0/≤10	NR/NR	100.00/95.24	N/A
I26 Pulmonary embolism	≤10/≤10	NR/NR	96.00/95.24	1.220 (0.076-19.507)
I46 Cardiac arrest	≤10/≤10	NR/NR	96.00/95.45	1.256 (0.078-20.155)
I60 Nontraumatic subarachnoid hemorrhage	0/0	NR/NR	100.00/100.00	N/A
I60-I62 Nontraumatic cerebral hemorrhage	≤10/0	NR/NR	95.00/100.00	N/A
I61 Nontraumatic intracerebral hemorrhage	≤10/0	NR/NR	95.00/100.00	N/A
I62 Other and unspecified nontraumatic intracranial hemorrhage	≤10/0	NR/NR	94.74/100.00	N/A
I63 Cerebral infarction	≤10/≤10	NR/NR	72.34/68.61	2.441 (0.399-14.942)
I73.9 Peripheral vascular disease	≤10/≤10	NR/NR	96.00/85.21	4.018 (0.417-38.679)
I82.4 Acute embolism and thrombosis of deep veins of lower extremity	≤10/≤10	NR/NR	83.33/84.42	4.943 (0.496-49.271)
M80 Osteoporosis with current pathological fracture	0/≤10	NR/NR	100.00/90.91	N/A
M81 Osteoporosis without current pathological fracture	≤10/≤10	NR/NR	66.67/85.71	N/A
Deceased	11/≤10	1429/1062	39.55/0.00	1.646 (0.691-3.920)

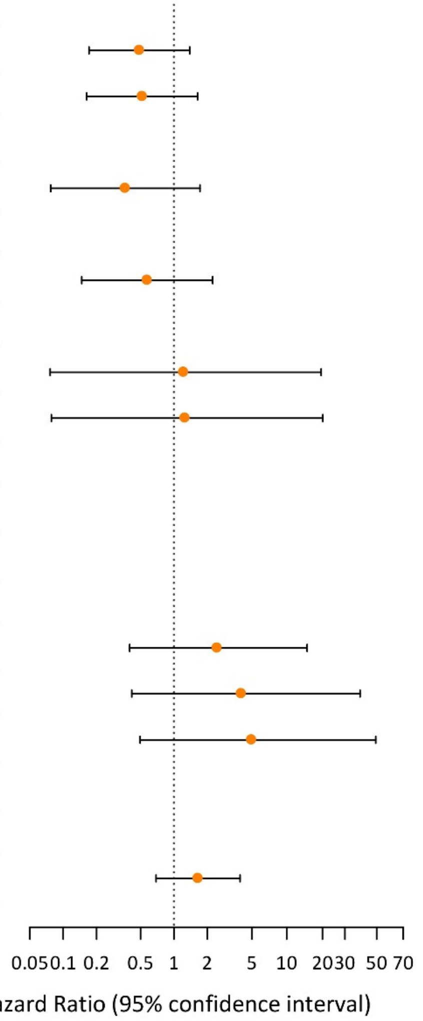

Supplementary Figure 2. Comparison of renal function outcomes and long-term vascular and skeletal events between patients with blood pH > 7.3 and pH ≤ 7.3, assessed between 1 week and 1 month postoperatively.

Outcome	Patients with outcome HCO3⁻ : >22/≤22	Median survival[1] (days) HCO3⁻ : >22/≤22	Survival probability[1] (%) HCO3⁻ : >22/≤22	Hazard Ratio (95% CI[2]) HCO3⁻ >22 as reference
eGFR ≤ 14 ml/min/1.73m^2	80/86	2678/3894	26.21/20.65	1.229 (0.906-1.667)
eGFR ≤ 10 ml/min/1.73m^2	54/66	NR/4180	55.00/22.88	1.402 (0.978-2.009)
eGFR ≤ 5 ml/min/1.73m^2	24/32	NR/NR	80.36/53.36	1.498 (0.881-2.547)
Creatinine ≥ 8 mg/dL	30/36	NR/NR	79.01/51.77	1.354 (0.833-2.200)
Creatinine ≥ 10 mg/dL	22/28	NR/NR	84.23/53.52	1.438 (0.822-2.516)
Hemodialysis	24/43	NR/5146	84.90/40.54	1.991 (1.207-3.284)
I21 Acute myocardial infarction	21/23	NR/NR	81.70/71.71	1.253 (0.693-2.266)
I26 Pulmonary embolism	14/15	NR/NR	85.39/91.42	1.160 (0.559-2.408)
I46 Cardiac arrest	≤10/≤10	NR/NR	95.57/96.48	2.199 (0.549-8.800)
I60 Nontraumatic subarachnoid hemorrhage	≤10/≤10	NR/NR	99.50/98.69	2.208 (0.200-24.350)
I60-I62 Nontraumatic cerebral hemorrhage	11/≤10	NR/NR	87.15/93.97	0.882 (0.365-2.131)
I61 Nontraumatic intracerebral hemorrhage	≤10/≤10	NR/NR	95.23/96.88	0.544 (0.164-1.809)
I62 Other and unspecified nontraumatic intracranial hemorrhage	≤10/≤10	NR/NR	90.98/95.70	1.618 (0.456-5.738)
I63 Cerebral infarction	19/24	NR/NR	87.08/74.36	1.422 (0.778-2.599)
I73.9 Peripheral vascular disease	16/23	NR/NR	87.31/71.00	1.631 (0.861-3.089)
I82.4 Acute embolism and thrombosis of deep veins of lower extremity	23/27	NR/NR	85.39/75.00	1.236 (0.708-2.156)
M80 Osteoporosis with current pathological fracture	≤10/≤10	NR/NR	95.77/99.47	0.156 (0.019-1.272)
M81 Osteoporosis without current pathological fracture	16/14	NR/NR	83.01/73.54	0.991 (0.482-2.038)
Deceased	71/94	3134/2236	24.58/18.29	1.455 (1.067-1.983)

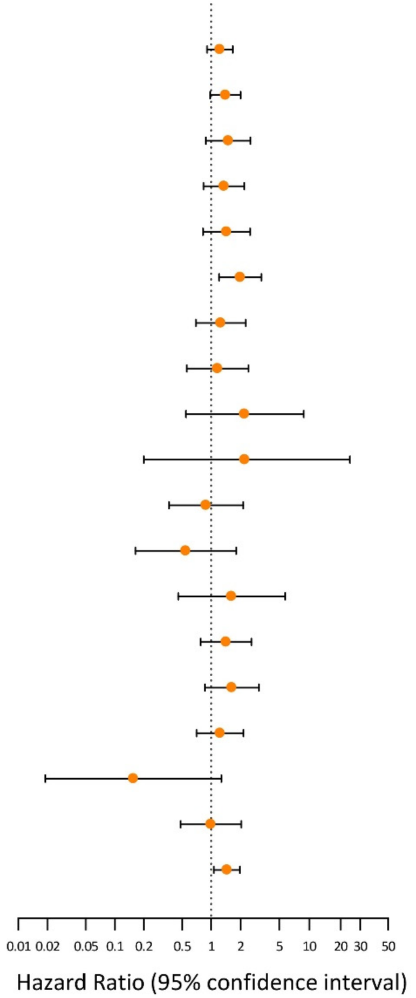

Supplementary Figure 3. Comparison of renal function outcomes and long-term vascular and skeletal events between patients with blood HCO3⁻ > 22 and HCO3⁻ ≤ 22 (mmol/L), assessed between 1 week and 1 month postoperatively.

Outcome	Patients with outcome Na+ : wnl/abn[3]	Median survival[1] (days) Na+ : wnl/abn[3]	Survival probability[1] (%) Na+ : wnl/abn[3]	Hazard Ratio (95% CI[2]) Na+ wnl as reference
eGFR ≤ 14 ml/min/1.73m^2	79/76	4177/3894	25.81/32.26	1.102 (0.804-1.511)
eGFR ≤ 10 ml/min/1.73m^2	56/50	NR/4891	55.77/38.02	1.049 (0.716-1.537)
eGFR ≤ 5 ml/min/1.73m^2	27/23	NR/NR	77.59/56.09	0.966 (0.553-1.688)
Creatinine ≥ 8 mg/dL	29/27	NR/NR	77.56/52.86	1.065 (0.630-1.801)
Creatinine ≥ 10 mg/dL	24/20	NR/NR	82.47/56.76	0.947 (0.523-1.716)
Hemodialysis	27/39	NR/4893	53.01/48.52	1.717 (1.050-2.808)
I21 Acute myocardial infarction	25/21	NR/NR	52.89/64.04	0.947 (0.530-1.693)
I26 Pulmonary embolism	16/21	NR/NR	81.47/89.52	1.473 (0.768-2.826)
I46 Cardiac arrest	≤10/≤10	NR/NR	90.00/87.02	2.019 (0.730-5.582)
I60 Nontraumatic subarachnoid hemorrhage	≤10/≤10	NR/NR	99.45/99.40	1.125 (0.070-17.987)
I60-I62 Nontraumatic cerebral hemorrhage	12/≤10	NR/NR	92.75/89.25	0.571 (0.214-1.526)
I61 Nontraumatic intracerebral hemorrhage	≤10/≤10	NR/NR	94.19/91.87	0.262 (0.056-1.217)
I62 Other and unspecified nontraumatic intracranial hemorrhage	≤10/≤10	NR/NR	96.92/97.84	0.895 (0.240-3.336)
I63 Cerebral infarction	19/22	NR/NR	87.26/71.62	1.366 (0.737-2.530)
I73.9 Peripheral vascular disease	16/20	NR/NR	88.12/80.20	1.439 (0.745-2.780)
I82.4 Acute embolism and thrombosis of deep veins of lower extremity	26/32	NR/NR	78.01/84.04	1.390 (0.828-2.333)
M80 Osteoporosis with current pathological fracture	≤10/≤10	NR/NR	91.93/86.67	1.336 (0.427-4.186)
M81 Osteoporosis without current pathological fracture	22/14	NR/NR	68.56/82.04	0.771 (0.393-1.512)
Deceased	71/91	3470/2483	28.25/0.00	1.538 (1.127-2.099)

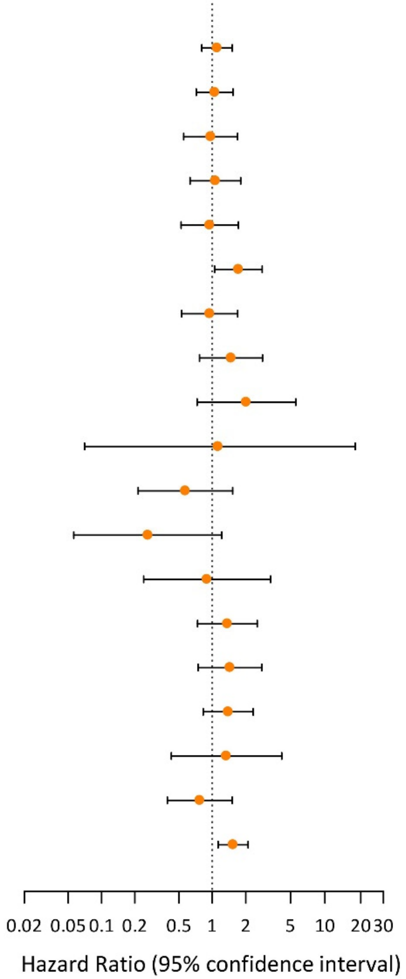

Supplementary Figure 4. Comparison of renal function outcomes and long-term vascular and skeletal events between patients with blood Na^+ wnl and Na^+ abn, assessed between 1 week and 1 month postoperatively.

Outcome	Patients with outcome K+ : wnl/abn[3]	Median survival[1] (days) K+ : wnl/abn[3]	Survival probability[1] (%) K+ : wnl/abn[3]	Hazard Ratio (95% CI[2]) K+ wnl as reference
eGFR ≤ 14 ml/min/1.73m^2	57/79	NR/1849	50.31/24.07	1.626 (1.155-2.288)
eGFR ≤ 10 ml/min/1.73m^2	41/58	NR/3933	58.29/29.56	1.629 (1.090-2.434)
eGFR ≤ 5 ml/min/1.73m^2	22/23	NR/NR	68.61/70.88	1.108 (0.616-1.993)
Creatinine ≥ 8 mg/dL	24/27	NR/NR	67.56/69.46	1.215 (0.700-2.111)
Creatinine ≥ 10 mg/dL	20/20	NR/NR	75.84/69.95	1.060 (0.568-1.976)
Hemodialysis	23/37	5146/NR	40.44/61.97	1.779 (1.056-3.000)
I21 Acute myocardial infarction	19/20	4314/NR	41.81/81.01	1.107 (0.588-2.083)
I26 Pulmonary embolism	11/16	NR/NR	86.67/87.99	1.576 (0.731-3.398)
I46 Cardiac arrest	≤10/≤10	NR/NR	82.12/86.66	2.413 (0.741-7.858)
I60 Nontraumatic subarachnoid hemorrhage	0/≤10	NR/NR	100.00/97.57	N/A
I60-I62 Nontraumatic cerebral hemorrhage	≤10/≤10	NR/NR	95.72/85.69	1.564 (0.556-4.394)
I61 Nontraumatic intracerebral hemorrhage	≤10/≤10	NR/NR	97.03/89.69	1.038 (0.260-4.152)
I62 Other and unspecified nontraumatic intracranial hemorrhage	≤10/≤10	NR/NR	98.06/94.43	2.112 (0.528-8.447)
I63 Cerebral infarction	14/19	NR/NR	84.30/72.46	1.532 (0.767-3.057)
I73.9 Peripheral vascular disease	15/15	NR/NR	68.39/86.73	1.049 (0.512-2.149)
I82.4 Acute embolism and thrombosis of deep veins of lower extremity	18/31	NR/NR	88.32/67.08	1.861 (1.041-3.329)
M80 Osteoporosis with current pathological fracture	≤10/≤10	NR/NR	95.25/96.98	0.870 (0.234-3.239)
M81 Osteoporosis without current pathological fracture	13/12	NR/NR	56.46/85.30	0.975 (0.444-2.143)
Deceased	64/84	2724/1825	22.99/0.00	1.452 (1.048-2.011)

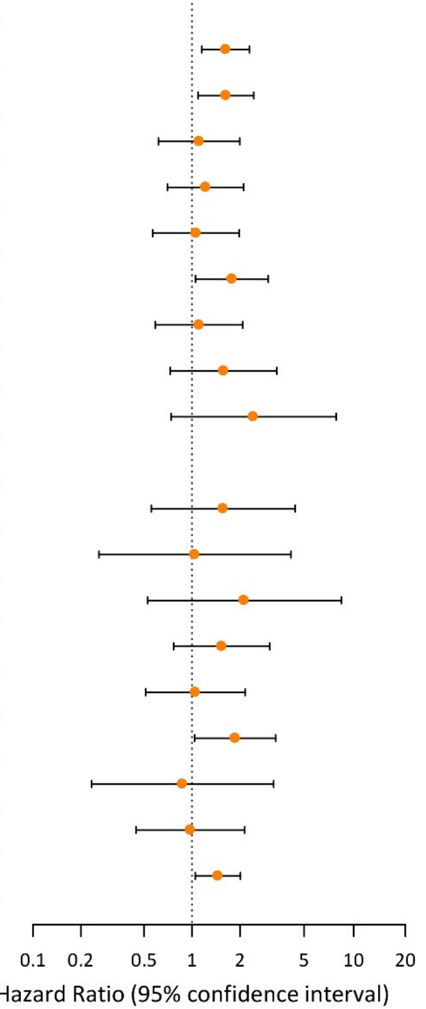

Supplementary Figure 5. Comparison of renal function outcomes and long-term vascular and skeletal events between patients with blood K+ wnl and K+ abn, assessed between 1 week and 1 month postoperatively.

Outcome	Patients with outcome Ca^{2+} : wnl/abn[3]	Median survival[1] (days) Ca^{2+} : wnl/abn[3]	Survival probability[1] (%) Ca^{2+} : wnl/abn[3]	Hazard Ratio (95% CI[2]) Ca^{2+} wnl as reference
eGFR ≤ 14 ml/min/1.73m^2	83/103	4297/2941	33.14/30.50	1.399 (1.047-1.869)
eGFR ≤ 10 ml/min/1.73m^2	60/73	NR/4811	56.25/38.34	1.371 (0.974-1.930)
eGFR ≤ 5 ml/min/1.73m^2	30/32	NR/NR	76.21/61.80	1.202 (0.730-1.979)
Creatinine ≥ 8 mg/dL	36/35	NR/NR	71.47/60.39	1.092 (0.685-1.739)
Creatinine ≥ 10 mg/dL	26/26	NR/NR	79.60/66.49	1.130 (0.656-1.948)
Hemodialysis	31/45	NR/NR	66.61/60.84	1.673 (1.058-2.645)
I21 Acute myocardial infarction	28/23	NR/NR	66.76/68.16	0.914 (0.526-1.588)
I26 Pulmonary embolism	≤10/29	NR/NR	93.78/84.57	5.486 (2.275-13.230)
I46 Cardiac arrest	≤10/≤10	NR/NR	90.31/90.50	1.507 (0.560-4.056)
I60 Nontraumatic subarachnoid hemorrhage	≤10/≤10	NR/NR	98.61/97.86	4.508 (0.503-40.382)
I60-I62 Nontraumatic cerebral hemorrhage	11/13	NR/NR	90.69/88.94	1.297 (0.581-2.900)
I61 Nontraumatic intracerebral hemorrhage	≤10/≤10	NR/NR	96.71/92.41	0.938 (0.315-2.796)
I62 Other and unspecified nontraumatic intracranial hemorrhage	≤10/≤10	NR/NR	93.95/95.42	1.447 (0.501-4.174)
I63 Cerebral infarction	21/26	NR/NR	86.11/75.56	1.446 (0.812-2.574)
I73.9 Peripheral vascular disease	22/22	NR/NR	85.34/74.95	1.123 (0.621-2.029)
I82.4 Acute embolism and thrombosis of deep veins of lower extremity	23/34	NR/NR	86.46/75.21	1.639 (0.965-2.784)
M80 Osteoporosis with current pathological fracture	≤10/≤10	NR/NR	96.09/91.99	1.144 (0.368-3.556)
M81 Osteoporosis without current pathological fracture	15/23	NR/NR	86.44/68.20	1.828 (0.951-3.515)
Deceased	86/108	3330/2236	26.27/22.47	1.452 (1.093-1.929)

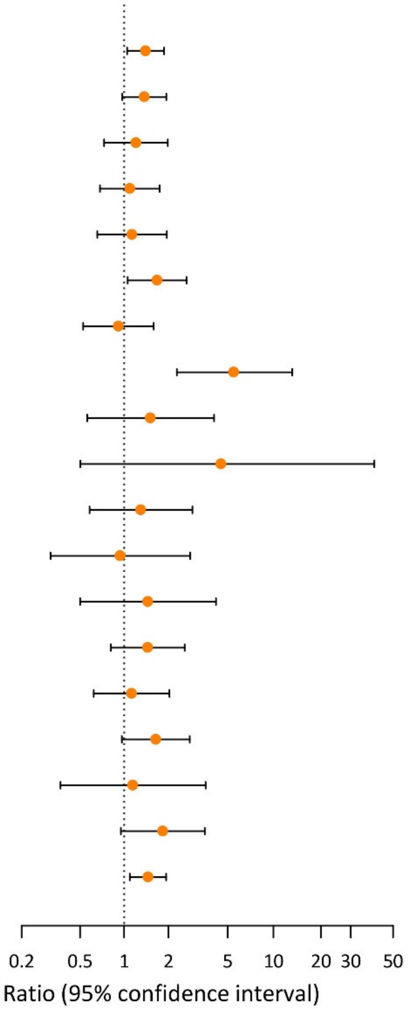

Supplementary Figure 6. Comparison of renal function outcomes and long-term vascular and skeletal events between patients with blood Ca^{2+} wnl and Ca^{2+} abn, assessed between 1 week and 1 month postoperatively.

Outcome	Patients with outcome Mg^{2+} : wnl/abn[3]	Median survival[1] (days) Mg^{2+} : wnl/abn[3]	Survival probability[1] (%) Mg^{2+} : wnl/abn[3]	Hazard Ratio (95% CI[2]) Mg^{2+} wnl as reference
eGFR ≤ 14 ml/min/1.73m[2]	62/61	2678/2941	31.77/31.54	1.090 (0.765-1.552)
eGFR ≤ 10 ml/min/1.73m[2]	44/39	4297/4891	46.53/40.59	0.947 (0.616-1.458)
eGFR ≤ 5 ml/min/1.73m[2]	20/18	NR/NR	77.96/57.56	0.963 (0.509-1.821)
Creatinine ≥ 8 mg/dL	24/18	NR/NR	75.67/56.05	0.794 (0.431-1.464)
Creatinine ≥ 10 mg/dL	20/12	NR/NR	74.83/60.32	0.630 (0.308-1.289)
Hemodialysis	25/26	NR/NR	55.20/52.13	1.164 (0.672-2.017)
I21 Acute myocardial infarction	22/11	NR/NR	65.45/91.56	0.533 (0.258-1.100)
I26 Pulmonary embolism	11/12	NR/NR	91.16/86.62	1.162 (0.512-2.633)
I46 Cardiac arrest	≤10/≤10	NR/NR	92.35/95.18	2.274 (0.567-9.117)
I60 Nontraumatic subarachnoid hemorrhage	≤10/≤10	NR/NR	96.48/98.37	1.155 (0.163-8.211)
I60-I62 Nontraumatic cerebral hemorrhage	11/≤10	NR/NR	83.97/94.42	0.607 (0.224-1.643)
I61 Nontraumatic intracerebral hemorrhage	≤10/≤10	NR/NR	88.01/96.83	0.411 (0.109-1.552)
I62 Other and unspecified nontraumatic intracranial hemorrhage	≤10/≤10	NR/NR	97.22/96.94	0.827 (0.185-3.697)
I63 Cerebral infarction	20/14	NR/NR	75.86/88.37	0.791 (0.399-1.568)
I73.9 Peripheral vascular disease	13/≤10	NR/NR	76.03/95.67	0.586 (0.234-1.469)
I82.4 Acute embolism and thrombosis of deep veins of lower extremity	12/23	NR/NR	88.50/67.83	2.172 (1.080-4.368)
M80 Osteoporosis with current pathological fracture	≤10/≤10	NR/NR	85.99/97.22	0.474 (0.122-1.835)
M81 Osteoporosis without current pathological fracture	16/≤10	NR/NR	75.55/72.27	0.701 (0.317-1.553)
Deceased	53/69	3655/1865	37.78/26.51	1.490 (1.042-2.133)

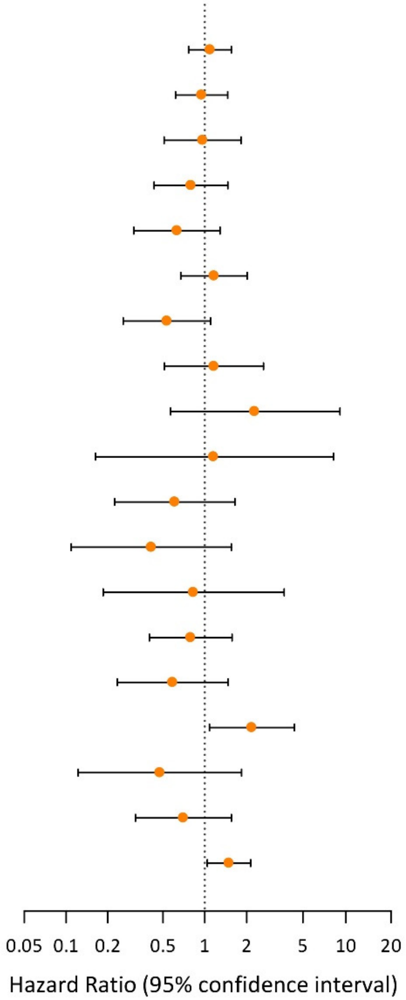

Supplementary Figure 7. Comparison of renal function outcomes and long-term vascular and skeletal events between patients with blood Mg^{2+} wnl and Mg^{2+} abn, assessed between 1 week and 1 month postoperatively.

Outcome	Patients with outcome P^+ : wnl/abn[3]	Median survival[1] (days) P^+ : wnl/abn[3]	Survival probability[1] (%) P^+ : wnl/abn[3]	Hazard Ratio (95% CI[2]) P^+ wnl as reference
eGFR ≤ 14 ml/min/1.73m^2	59/78	4866/1849	42.73/30.34	1.746 (1.243-2.452)
eGFR ≤ 10 ml/min/1.73m^2	45/55	4891/2884	48.87/37.26	1.607 (1.082-2.387)
eGFR ≤ 5 ml/min/1.73m^2	25/25	NR/NR	66.21/56.99	1.289 (0.739-2.249)
Creatinine ≥ 8 mg/dL	29/29	NR/4221	65.78/48.84	1.277 (0.762-2.140)
Creatinine ≥ 10 mg/dL	24/20	NR/NR	66.44/60.83	1.093 (0.602-1.983)
Hemodialysis	25/40	NR/NR	57.86/52.73	2.114 (1.279-3.496)
I21 Acute myocardial infarction	22/15	NR/NR	52.87/76.45	0.886 (0.459-1.712)
I26 Pulmonary embolism	11/15	NR/NR	88.74/87.40	1.674 (0.766-3.659)
I46 Cardiac arrest	≤10/≤10	NR/NR	87.27/91.89	3.126 (0.953-10.247)
I60 Nontraumatic subarachnoid hemorrhage	≤10/≤10	NR/NR	99.17/98.44	2.486 (0.225-27.434)
I60-I62 Nontraumatic cerebral hemorrhage	≤10/≤10	NR/NR	91.05/80.89	1.761 (0.651-4.763)
I61 Nontraumatic intracerebral hemorrhage	≤10/≤10	NR/NR	93.56/93.83	2.875 (0.712-11.604)
I62 Other and unspecified nontraumatic intracranial hemorrhage	≤10/≤10	NR/NR	97.34/83.73	1.648 (0.439-6.183)
I63 Cerebral infarction	11/18	NR/NR	85.05/80.64	2.206 (1.036-4.700)
I73.9 Peripheral vascular disease	12/17	NR/NR	80.45/83.51	1.822 (0.868-3.824)
I82.4 Acute embolism and thrombosis of deep veins of lower extremity	12/32	NR/NR	88.76/57.51	3.374 (1.732-6.570)
M80 Osteoporosis with current pathological fracture	≤10/≤10	NR/NR	98.29/94.80	3.285 (0.636-16.959)
M81 Osteoporosis without current pathological fracture	17/11	NR/NR	64.88/80.25	0.917 (0.425-1.980)
Deceased	68/84	3171/1713	27.79/0.00	1.716 (1.244-2.367)

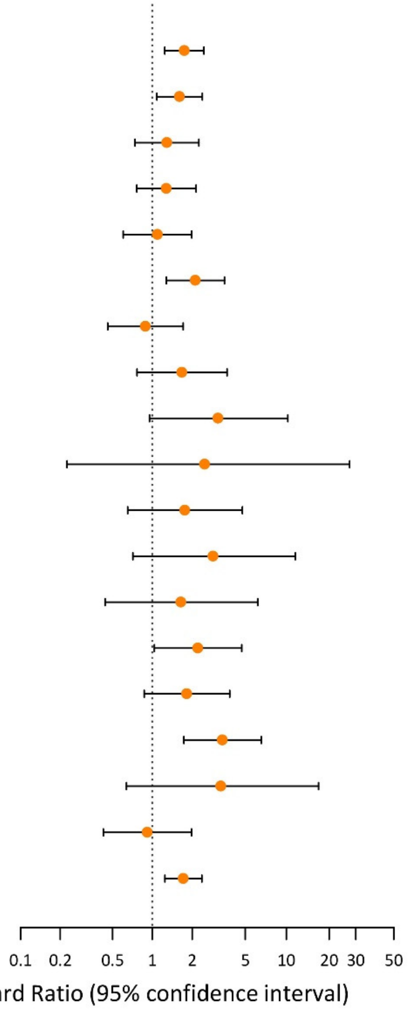

Supplementary Figure 8. Comparison of renal function outcomes and long-term vascular and skeletal events between patients with blood P wnl and P abn, assessed between 1 week and 1 month postoperatively.
